# Patterns of Telemedicine Use and the Digital Divide Among Adults in Poland’s Postpandemic Era: Nationwide Cross-Sectional Survey

**DOI:** 10.2196/88442

**Published:** 2026-09-22

**Authors:** Aleksander Biesiada, Anna Zimny-Zając, Karolina Kłoda, Mateusz Babicki, Agnieszka Mastalerz-Migas, Beata Jankowska-Polańska, Siddarth Agrawal

**Affiliations:** 1 Polish Society of Family Medicine, Syrokomli 1, Wrocław, Lower Silesia, 51-141, Poland, 48 71-32-66-870, 48 71-325-43-41; 2 Faculty of Health Science, Institute of Public Health, Medical College, Jagiellonian University, Kraków, Lesser Poland, Poland; 3 Medonet, Ringier Axel Springer Poland, Warszawa, Mazowieckie, Poland; 4 Katedra i Zakład Medycyny Rodzinnej, Wroclaw Medical University, Wrocław, Lower Silesia, Poland; 5 Faculty of Medicine, Wrocław University of Science and Technology, Wrocław, Lower Silesia, Poland; 6 Labplus R&D, Wrocław, Lower Silesia, Poland

**Keywords:** telemedicine, health care access, eHealth, e-prescriptions, teleconsultations, digital exclusion

## Abstract

**Background:**

The COVID-19 pandemic transformed telemedicine in Poland from a niche service into a core mode of health care delivery, supported by nationwide eHealth infrastructure such as obligatory e-prescriptions and the central P1 platform. Despite this rapid expansion, concerns persist that the benefits of digital health are not shared equally, and that a digital divide limits equitable access to telemedicine services across sociodemographic and geographic groups.

**Objective:**

This study aimed to describe the patterns of telemedicine and eHealth tool use among Polish adults in the postpandemic era, to identify key sociodemographic, economic, and health-related predictors of their adoption, and to map digital inequalities in access to health care services.

**Methods:**

This cross-sectional study used data from the “National Test for Poles’ Health” (Narodowy Test Zdrowia Polaków), a nationwide online survey conducted annually in Poland between 2020 and 2024. The full surveyed sample comprised 1,196,102 adults, of whom 820,000 completed the telemedicine-use module and formed the analytic sample; detailed questions on 12 specific eHealth services were administered to a randomly assigned subsample of 120,000 respondents under a split-questionnaire design. Sociodemographic factors (age, gender, education level, and place of residence), health status, and barriers to accessing care were analyzed as predictors using ordered and binary logistic regression models, with results expressed as odds ratios (ORs) with 95% CIs, and statistical significance set at *P*≤.05.

**Results:**

Overall, 49.7% (407,502/820,000) of respondents reported using a telemedicine visit at least once in the preceding 12 months. Telephone consultations were the most common form of remote visit (43,598/120,000, 36.3%), whereas video consultations were rare (8736/120,000, 7.3%). System-driven tools showed high adoption (e-prescriptions: 87,121/120,000, 72.6%; e-referrals: 64,083/120,000, 53.4%), while patient-driven tools, such as mobile health apps (24,341/120,000, 20.3%), were used less frequently. Education and place of residence were the strongest sociodemographic predictors: participants with a master’s degree were 2.2 times more likely than those with secondary education to use the Internet Patient Account (OR 2.2, 95% CI 2.1‐2.2), and residents of cities with over 500,000 inhabitants had higher odds of telemedicine use (OR 1.5, 95% CI 1.5‐1.6). Chronic disease was a strong health-related predictor (OR 1.8, 95% CI 1.7‐1.9). Gender differences were also observed: women used telephone consultations more often, whereas men more frequently used the Internet Patient Account.

**Conclusions:**

Telemedicine adoption in postpandemic Poland is driven more by pre-existing socioeconomic advantage, particularly higher education and urban residence, than by clinical need alone, indicating a persistent digital divide. Telemedicine functions largely as a tool of convenience for the already connected rather than a tool of necessity for underserved groups. Targeted interventions, including community-based digital literacy programs, investment in rural broadband infrastructure, and guaranteed hybrid in-person alternatives, are needed to ensure that Poland’s eHealth transformation promotes inclusion rather than exacerbating health inequalities.

## Introduction

### Problem

The COVID-19 pandemic acted as a powerful catalyst for the global adoption of telemedicine, transforming it from a niche service into a cornerstone of health care delivery [1]. Digital technologies became central to pandemic planning and response worldwide, and health systems that could rapidly scale remote care were better able to maintain continuity of services during successive waves of infection [2]. Poland serves as a compelling case study in this rapid transition. The country entered the pandemic with key eHealth infrastructure already in place, including a nationwide e-prescription system (obligatory from January 2020) and the central P1 platform for e-referrals and patient records [3,4]. This foundation enabled a swift pivot to remote care, with teleconsultations surging to 58.6 million in 2020 and remaining substantial in the postpandemic era [5].

Despite this technological progress, there are significant concerns that the benefits of digital health are not being shared equally. While 95.9% of Polish households had internet access in 2023, digital literacy remains a major barrier, particularly among older populations: only 13.7% of individuals aged 65 to 74 years possess basic digital skills [6]. This digital divide is compounded by a rural-urban gap in internet usage and the fact that only half of Poles use the internet to seek health information [7,8]. The World Health Organization (WHO) has warned that digital transformation, if not accompanied by deliberate equity-oriented policies, may deepen rather than reduce health inequalities, and has called on member states to monitor equitable access as a core element of national digital health strategies [9]. Contemporary digital divide research likewise emphasizes that inequalities extend beyond physical access to technology (the first-level divide) to differences in digital skills and patterns of use (the second-level divide) and, ultimately, to the tangible benefits that individuals derive from being online (the third-level divide) [10]. Within health care, these mechanisms have been conceptualized as digital determinants of health—including digital literacy, access to technological resources, and trust in digital systems—that interact with wider social determinants to shape whether patients can actually benefit from services such as teleconsultations or patient portals [11]. This raises a critical question: is the rapid digitalization of health care inadvertently widening pre-existing health inequalities?

### Review of Relevant Scholarship

The promise of telemedicine lies in its potential to overcome traditional barriers to care. Tools such as teleconsultations—real-time virtual appointments via video or phone—are intended to improve access for patients in remote locations or with mobility challenges [12-14]. Similarly, digital solutions like e-prescriptions and e-referrals, which streamline communication between providers, pharmacies, and specialists, are designed to increase efficiency and convenience for all patients. Meta-analyses have confirmed the clinical potential of these tools, particularly for improving outcomes in chronic conditions like diabetes [15]. However, the effective realization of these benefits is contingent upon equitable access and patient adoption.

International evidence accumulated before and during the pandemic consistently indicates that telemedicine adoption follows a social gradient. In the United States, analyses of the nationally representative Health Information National Trends Survey demonstrated that older age, lower education, and lower income were associated with substantially lower engagement in eHealth activities well before COVID-19 [16]. During the pandemic, Luo et al [17] found that videoconference visits were concentrated among patients with private insurance and a college education, and a national study estimated that 38% of US adults aged 65 years or older were not ready for video visits, chiefly because of inexperience with technology [18]. Digital access has consequently been described as an emerging social determinant of health in its own right [19]. Notably, however, the direction of these associations is not universal: a nationwide study from the United Arab Emirates reported higher telemedicine use among older adults [20], suggesting that the shape of the digital divide is context-dependent and must be characterized empirically within each health system.

Similar patterns have been described in European settings. A systematic review of eHealth use among people with chronic diseases—the group with potentially the most to gain from remote care—identified higher age, lower income, lower education, living alone, and rural residence as consistent correlates of lower eHealth use [21]. Reviews of telemedicine and digital health equity likewise conclude that, in the absence of deliberate countermeasures, the digitalization of health care tends to reproduce existing patterns of exclusion affecting older adults, less-educated groups, and rural populations [22].

Evidence from Poland remains comparatively sparse and fragmented. Earlier national surveys documented a steady increase in the use of the internet for health purposes, together with marked sociodemographic differences in uptake [23]. Studies conducted during the pandemic focused mainly on patients’ satisfaction with and attitudes toward teleconsultations [3,24], while Lubomski et al [25] examined the acceptance of teleconsultations among 2318 patients and health care professionals without analyzing sociodemographic determinants. To date, no large-scale, population-based study has systematically quantified how sociodemographic and health-related factors predict the use of a comprehensive range of eHealth services in Poland, and little is known about which patterns of use have persisted once the emergency conditions of the pandemic subsided.

### Hypothesis, Aims, and Objectives

This gap is consequential for policy. Poland’s eHealth transformation is among the most extensive in Central and Eastern Europe, and decisions about its further development—including investments in digital literacy, broadband infrastructure, and the regulation of teleconsultation standards in primary care—require population-level evidence on who uses these services and who is left behind [4,5]. Therefore, this study leverages a uniquely large, nationwide dataset to address this critical gap. Our primary aim is to characterize the prevalence of use of 12 distinct telemedicine and eHealth tools among Polish adults in the postpandemic era. Furthermore, we seek to identify the key sociodemographic, economic, and health-related predictors of their adoption. By doing so, this research maps the contours of digital inequality in access to Polish health care, providing an evidence base to guide targeted policy interventions aimed at ensuring that the benefits of eHealth are accessible to all.

This divide can be understood through the Digital Health Equity Framework, which posits that socioeconomic contexts (like education and urbanicity) directly influence digital determinants of health (such as access, literacy, and trust) [11]. Based on this framework, we hypothesized that (1) eHealth adoption in postpandemic Poland is driven more by socioeconomic status than clinical need; and (2) telemedicine currently functions as a ’tool of convenience’ for the already connected, rather than a “tool of necessity” for the isolated.

## Methods

### Inclusion and Exclusion Criteria

The study population consisted of adult Polish internet users aged 18 years or older who participated in the “Narodowy Test Zdrowia Polaków” (National Test for Poles’ Health) between 2020 and 2024. All respondents who completed the core sociodemographic and health-status items were retained for the descriptive analyses, forming the full surveyed sample (N=1,196,102; Multimedia Appendix 1). The regression analyses were restricted to respondents with valid data on the relevant outcome: the general telemedicine-use model was fitted on the 820,000 respondents who completed the telemedicine-use module, and the service-specific models on a randomly assigned subsample of 120,000 respondents who received the detailed eHealth-service items under a split-questionnaire design (see the “Sample Size, Power, and Precision” section).

### Participant Characteristics

The full surveyed sample comprised 1,196,102 adult participants and is characterized in Multimedia Appendix 1. Participant characteristics included age, gender, highest level of education, and place of residence. Health-related characteristics included diagnosed chronic and long-term diseases, including specific conditions such as hypertension and depression, as well as self-assessed general physical and mental health on a 5-point scale.

### Sampling Procedures

The survey was distributed via Medonet, a major Polish health-focused online platform, to achieve a broad and diverse reach. Recruitment involved open, voluntary participation through online advertisements and articles on the Medonet platform and its associated channels. A total of 1,196,102 participants completed the survey’s core sociodemographic and health-status items and constitute the full surveyed sample described in Multimedia Appendix 1; of these, 820,000 (68.6%) also completed the telemedicine-use module. The composition of the analytic sample and of the split-questionnaire subsample is detailed under the “Sample Size, Power, and Precision” section.

### Ethical Considerations

This study was conducted in accordance with the ethical principles of the Declaration of Helsinki (1964 and its later amendments). The research protocol for this survey-based study, which involved the collection of anonymized data and voluntary participation, was reviewed by the Bioethics Committee of the Military Chamber of Physicians (Wojskowa Izba Lekarska) in Warsaw, Poland; the committee determined that formal ethical approval was not required for this type of study, given its anonymized and voluntary nature (decision number: KB 65/2024). All participants provided informed consent electronically by accepting the study’s terms and conditions before beginning the questionnaire, and this consent covered the research use of the collected data, including the analyses reported here; no additional consent was required for the present analyses because the dataset was fully anonymized before being made available to the research team. Privacy and confidentiality were protected throughout the study: the dataset was fully anonymized, no personally identifiable information was available to the researchers, and no identifiable participant information is included in this manuscript or its supplementary materials. Participation was entirely voluntary, and no compensation of any type or amount was provided to participants. Finally, the manuscript and supplementary materials contain no images of individual participants; therefore, identification of individual participants is not possible.

### Sample Size, Power, and Precision

The full survey reached 1,196,102 participants, who constitute the surveyed sample characterized in Multimedia Appendix 1. The telemedicine-use module was completed by 820,000 respondents, who constitute the analytic sample for the general telemedicine-use (ordered logistic) model. Detailed questions on the 12 specific eHealth services were presented to a randomly assigned subsample of 120,000 respondents under a split-questionnaire design used in the relevant survey wave; these respondents constitute the analytic subsample for the service-specific (binary logistic) models. No interim analyses or stopping rules were applied. Given the large sample size, precision was assessed using 95% CIs and effect-size estimates.

### Measures and Covariates

The self-administered online questionnaire collected data across several key domains. The primary outcome variables assessed the self-reported use of 12 distinct eHealth services within the preceding 12 months, with responses for each service coded dichotomously as “Yes” or “No.” These services included remote visits via video or telephone, e-prescriptions, e-referrals, and the national Internet Patient Account, among others. Independent variables included sociodemographic factors such as age, gender, highest level of education, and place of residence. Health status was evaluated through questions on the presence of diagnosed chronic and long-term diseases, including specific conditions such as hypertension and depression, as well as self-assessed general physical and mental health on a 5-point scale. Potential barriers to health care were measured by inquiring about forgoing medical services for financial reasons and the self-reported ability to arrange transportation to appointments.

### Data Collection

Data were collected using a self-administered online questionnaire. The survey was completed remotely by participants through the Medonet platform and its associated online channels. Data collection took place annually in Poland between 2020 and 2024. To prevent multiple entries from the same individual, the survey platform used IP address tracking and browser cookies. All participants who completed the telemedicine and health status modules provided full responses to the survey items; therefore, there were no missing data in this study.

### Quality of Measurements, Instrumentation, and Masking

The questionnaire items assessing the use of 12 eHealth services were developed specifically for the National Test for Poles’ Health. As these items relied on self-report over a 12-month recall period, this introduces a potential for recall bias, which is addressed in the limitations.

### Psychometrics

The questionnaire items assessing the use of eHealth services were developed specifically for this survey and were not designed as composite psychometric scales. Self-assessed physical and mental health were measured using single-item ordinal measures; therefore, internal consistency could not be estimated.

### Conditions and Design

This study employed a cross-sectional design, analyzing pooled data from the “Narodowy Test Zdrowia Polaków” (National Test for Poles’ Health). This is a large-scale, nationwide online health survey conducted annually in Poland between 2020 and 2024. The survey is designed to gather comprehensive data on the health status, lifestyle, and health care use of the Polish population. No experimental conditions were manipulated. All variables were naturally observed. Reporting follows the APA Journal Article Reporting Standards for Quantitative Research (JARS-Quant) [26]. According to the JARS-Quant framework, this study is classified as a nonexperimental observational cross-sectional study.

### Data Diagnostics

Before analysis, the dataset was screened to identify participants who did not complete the telemedicine-use module; these participants were retained in the descriptive analyses (Multimedia Appendix 1) but excluded from the regression analytic sample. No further post–data-collection exclusion criteria were applied. All participants included in the final analytic sample had complete data for the analyzed variables; therefore, no imputation of missing data was performed. Because the main outcomes and covariates were categorical or ordinal, statistical outlier detection was not applicable. Data distributions were examined descriptively using frequencies and percentages. No data transformations were applied.

### Analytic Strategy

Descriptive statistics were used to summarize the characteristics of the study sample. The chi-square test of independence was used for bivariate comparisons between genders, with effect sizes, φ or Cramer *V*, calculated due to the large sample size. To identify factors influencing the adoption of eHealth services, a series of logistic regression models were applied. Ordered logistic regression was used for the overall multilevel telemedicine use variable, while binary logistic regression was used for the dichotomous outcomes for each of the 12 specific tools. Results are presented as odds ratios (OR) with 95% CIs. All analyses were performed using Statistica version 13.3 (TIBCO Software Inc), and a *P* value ≤.05 was considered significant.

## Results

### Participant Flow

Of 1,196,102 respondents included in the overall survey dataset, 820,000 completed the telemedicine-use module and constituted the main analytic sample for analyses of telemedicine use. Analyses of specific eHealth tools were conducted in a subsample of 120,000 respondents, as indicated in the relevant tables.

### Recruitment

Data were collected between 2020 and 2024. The study was cross-sectional; therefore, no repeated measurements or follow-up assessments were conducted.

### Characteristics of the Surveyed Sample

Multimedia Appendix 1 summarizes the characteristics of the full surveyed sample (N=1,196,102), that is, all respondents who completed the core sociodemographic and health-status items; the analytic samples used for the regression models are smaller and are defined in the “Methods” section (n=820,000 for general telemedicine use; n=120,000 for the service-specific models). The surveyed sample was predominantly female (700,408/1,196,102, 58.6%), with a mean age of 51.8 (SD 14.9) years. The most common place of residence was a rural area (252,448/1,196,102, 21.1%), and the most frequent level of education was a master’s degree (480,486/1,196,102, 40.2%). Over half of the respondents (669,615/1,196,102, 56%) reported having at least one chronic or long-term disease. While most participants rated their general physical (504,212/1,196,102, 42.2%) and mental (511,182/1,196,102, 42.7%) health as “good,” women were significantly more likely than men to report “average,” “bad,” or “very bad” health in both domains. It is important to note that due to the exceptionally large sample size, bivariate chi-square tests yielded highly significant results (*P*<.001) for virtually all demographic and health-related comparisons. However, the calculated effect sizes (Cramer *V* and φ coefficients) were generally very weak, ranging from 0.02 to 0.17. This indicates that while the observed differences between genders and demographic groups are statistically significant, their practical or clinical magnitude may be limited, underscoring the necessity of interpreting these differences in the context of the large dataset.

### Prevalence and Patterns of Telemedicine Use

Overall, just under half of the participants (407,502/820,000, 49.7%) reported using a telemedicine visit at least once in the past 12 months. However, the adoption of specific eHealth tools was assessed in a subsample of 120,000 respondents and varied significantly (Table 1). Telephone-based services were the most common form of remote consultation, used by 43,598 out of 120,000 (36.3%) respondents, whereas video consultations were rare (8736/120,000, 7.3%). Foundational eHealth tools like e-prescriptions (87,121/120,000, 72.6%) and e-referrals (64,083/120,000, 53.4%) showed high rates of adoption. In contrast, more interactive or patient-driven tools, such as mobile health apps (24,341/120,000, 20.3%) and email consultations with a doctor (12,880/120,000, 10.7%), were used less frequently.

Regarding patient attitudes (Table 2), the majority of those who used a telemedicine visit found that it met their expectations either completely (119,199/407,502, 29.3%) or partially, though they still preferred traditional visits (152,032/407,502, 37.3%). Only 44,801 out of 407,502 (11%) stated that the visit completely failed to meet their expectations. Among those who did not use telemedicine, the most common reason was a perceived lack of need (277,275/412,498, 67.2%), followed by a preference for traditional in-person visits (64,297/412,498, 15.6%).

**Table 1.** Use of telemedicine services by gender among adult participants in a nationwide cross-sectional online survey in Poland, 2020‐2024 (n=820,000 for general use; n=120,000 subsample for specific services). Values are n (%) of the total sample.

	Total, n (%)	Women, n (%)	Men, n (%)	Women-Men difference (*P* value; effect size)
Use of a telemedicine visit in the past 12 months				*P*<.001; *V*_c_^a^=0.12
Many times	146,581 (17.9)	98,984 (12.1)^b^	47,597 (5.8)	
At least once	260,921 (31.8)	162,170 (19.8)^b^	98,751 (12)	
No	412,498 (50.3)	220,127 (26.8)	192,371 (23.5)^b^	
Use of the following forms of remote (eHealth or telemedicine) services or applications in the past 12 months				
Remote visit (online consultation and televising) via video conferencing system				*P*=.08; φ=−0.005
Yes	8736 (7.3)	5401 (4.5)	3335 (2.8)	
No	111,264 (92.7)	69,849 (58.2)	41,415 (34.5)	
Remote visit (online consultation and televising) via telephone				*P*<.001; φ=0.06
Yes	43,598 (36.3)	29,072 (24.2)^b^	14,526 (12.1)	
No	76,402 (63.7)	46,178 (38.5)	30,224 (25.2)^b^	
E-prescriptions				*P*<.001; φ=0.02
Yes	87,121 (72.6)	55,190 (46)^b^	31,931 (26.6)	
No	32,879 (27.4)	20,060 (16.7)	12,819 (10.7)^b^	
E-referrals				*P*<.001, φ=0.02
Yes	64,083 (53.4)	40,671 (33.9)^b^	23,412 (19.5)	
No	55,917 (46.6)	34,579 (28.8)	21,338 (17.8)^b^	
eHealth care certificates				*P*<.001; φ=0.03
Yes	20,002 (16.7)	13,102 (10.9)^b^	6900 (5.8)	
No	99,998 (83.3)	62,148 (51.8)	37,850 (31.5)^b^	
E-registrations				*P*<.001; φ=−0.004
Yes	43,403 (36.2)	26,041 (21.7)	17,362 (14.5)^b^	
No	76,597 (63.8)	49,209 (41)^b^	27,388 (22.8)	
Internet Patient Account (available at patient.gov.pl)				*P*<.001; φ=−0.005
Yes	59,520 (49.6)	35,784 (29.8)	23,736 (19.8)^b^	
No	60,480 (50.4)	39,466 (32.9)^b^	21,014 (17.5)	
A website with test results (eg, imaging and laboratory results)				*P*<.001; φ=0.02
Yes	64,214 (53.5)	40,873 (34.1)^b^	23,341 (19.5)	
No	55,786 (46.5)	34,377 (28.6)	21,409 (17.8)^b^	
A mobile app that supports a healthy lifestyle, such as activity				*P*<.001; φ=−0.04
Yes	24,341 (20.3)	14,380 (12)	9961 (8.3)^b^	
No	95,659 (79.7)	60,870 (50.7)^b^	34,789 (29)	
Email for advice from a doctor or other employee				*P*<.001; φ=−0.05
Yes	12,880 (10.7)	7110 (5.9)	5770 (4.8)^b^	
No	107,120 (89.3)	68,140 (56.8)^b^	38,980 (32.5)	
Portal or website offering paid health advice				*P*<.001; φ=−0.04
Yes	9715 (8.1)	5534 (4.6)	4181 (3.5)^b^	
No	110,285 (91.9)	69,716 (58.1)^b^	40,569 (33.8)	
A portal or website offering the possibility to issue a prescription				*P*<.001; φ=−0.03
Yes	8089 (6.7)	4710 (3.9)	3379 (2.8)^b^	
No	111,911 (93.3)	70,540 (58.8)^b^	41,371 (34.5)	

a Cramer *V*.

b Surpluses for chi-square independence test.

c Percentages in the “Women” and “Men” columns are calculated with the total sample as the denominator. Because the sample is predominantly female, within-gender percentages differ (eg, for the Internet Patient Account: 47.6% of women vs 53.0% of men). The direction of gender differences after adjustment is given by the odds ratios reported in Multimedia Appendix 2.

**Table 2.** Attitudes towards telemedicine by gender, among the 820,000-respondent analytic sample, nationwide cross-sectional online survey, Poland, 2020‐2024.

	Total, n (%)	Women, n (%)	Men, n (%)	Women-men difference (*P* value; effect size)
Did the telemedicine visit meet your expectations?^a^				*P*<.001; *V*_c_^b^=0.01
Yes, it completely met my expectations	119,199 (29.3)	76,876 (18.9)^c^	42,323 (10.4)	
Yes, but I prefer traditional visits to the doctor’s office	152,032 (37.3)	96,460 (23.7)	55,572 (13.6)^c^	
Only partially met my expectations	91,470 (22.4)	59,248 (14.5)^c^	32,222 (7.9)	
No, it completely failed to meet my expectations	44,801 (11)	28,570 (7)	16,231 (4)^c^	
Why did you not use a telemedicine visit?^d^				*P*<.001; *V*_c_=0.06
There was no need for it	277,275 (67.2)	144,098 (34.9)	133,177 (32.3)^c^	
My health problem was too serious to use a telemedicine visit	10,170 (2.5)	6643 (1.6)^c^	3527 (0.9)	
Don’t know how to use telemedicine visits	37,158 (9)	19,768 (4.8)	17,390 (4.2)^c^	
I prefer a classic visit to a doctor	64,297 (15.6)	37,395 (9.1)^c^	26,902 (6.5)	
Have no confidence in telemedicine services	23,598 (5.7)	12,223 (3)	11,375 (2.8)^c^	

a Percentages are calculated based on the subgroup of respondents for whom the telemedicine visit met their expectations (n=407,502).

b Vc: Cramer *V*.

c Surpluses for chi-square independence test.

d Percentages are calculated based on the subgroup of respondents who did not used a telemedicine visit (n=412,498).

### Predictors of Telemedicine Adoption

Logistic regression analyses revealed significant and consistent sociodemographic disparities in the adoption of nearly all eHealth services (Multimedia Appendix 3 and Table S1 in Multimedia Appendix 2). Table 3 summarizes the barriers to the use of medical services and difficulties in reaching a doctor in the full surveyed sample.

**Table 3.** Barriers to the use of medical services and difficulties in reaching a doctor in the full surveyed sample (N=1,196,102), nationwide cross-sectional online survey, Poland, 2020‐2024. Values are n (%) of the total sample.

	Total, n (%)	Women, n (%)	Men, n (%)	Women-men difference (*P* value; effect size)
Have you ever given up for financial reasons				
A visit to the doctor				*P*<.001; *V*_c_^a^=0.17
Yes	225,340 (18.8)	170,443 (14.2)^b^	54,897 (4.6)	
No	555,675 (46.5)	296,516 (24.8)	259,159 (21.7)^b^	
Have never been put in this situation	415,087 (34.7)	233,449 (19.5)	181,638 (15.2)^b^	
Dental visits				*P*<.001; *V*_c_=0.14
Yes	283,550 (23.7)	201,513 (16.8)^b^	82,037 (6.9)	
No	540,764 (45.2)	292,285 (24.4)	248,479 (20.8)^b^	
Have never been put in this situation	371,788 (31.1)	206,610 (17.3)	165,178 (13.8)^b^	
Purchasing a prescription drug				*P*<.001; *V*_c_=0.11
Yes	154,775 (12.9)	111,924 (9.4)^b^	42,851 (3.6)	
No	625,506 (52.3)	349,555 (29.2)	275,951 (23.1)^b^	
Have never been put in this situation	415,821 (34.8)	238,929 (20)	176,892 (14.8)^b^	
Purchasing needed medical equipment				*P*<.001; *V*_c_=0.12
Yes	118,411 (9.9)	86,258 (7.2)^b^	32,153 (2.7)	
No	460,169 (38.5)	241,565 (20.2)	218,604 (18.3)^b^	
Have never been put in this situation	617,522 (51.6)	372,585 (31.1)^b^	244,937 (20.5)	
Are you able to provide transportation to your medical appointment?				*P*<.001; *V*_c_=0.16
Yes, without any difficulty	959,202 (80.2)	523,586 (43.8)	435,616 (36.4)^b^	
Yes, but with some difficulties	112,456 (9.4)	82,817 (6.9)^b^	29,639 (2.5)	
Yes, but with great difficulties	17,958 (1.5)	13,524 (1.1)^b^	4434 (0.4)	
Not able to provide transportation to the place of medical appointment	9674 (0.8)	7319 (0.6)^b^	2355 (0.2)	
Do not need transportation to the place of medical appointment—I walk there	70,233 (5.9)	52,664 (4.4)^b^	17,569 (1.5)	
Difficult to say	26,579 (2.2)	20,498 (1.7)^b^	6081 (0.5)	

a Cramer *V*.

b Surpluses for chi-square independence test.

### Sociodemographic Factors

Higher education and urban residence were among the strongest predictors of use. For instance, participants with a master’s degree were 1.7 times more likely to use e-prescriptions (Table S1 in Multimedia Appendix 2, model 3) and 2.2 times more likely to use the Internet Patient Account (Table S1 in Multimedia Appendix 2, model 7) than those with a secondary education. Similarly, residents of large cities (>500,000 inhabitants) had significantly higher odds of using overall telemedicine visits (OR 1.5, 95% CI 1.5‐1.6) and e-referrals (OR 1.4, 95% CI 1.3‐1.4; Table S1 in Multimedia Appendix 4, model 1). Conversely, having a primary or lower secondary education was a consistent and powerful barrier, associated with a 40%‐60% lower likelihood of using foundational tools like e-prescriptions and the Internet Patient Account.

### Health Status

The presence of a chronic disease was a major driver of telemedicine adoption. Individuals with long-term health conditions were 1.8 times more likely to have had a telemedicine visit and twice as likely to use e-prescriptions. Specific conditions, such as a depression diagnosis, were notably associated with the use of services requiring more patient engagement, like remote video consultations (OR 1.5, 95% CI 1.4‐1.5) and online prescription portals (OR 1.4, 95% CI 1.3‐1.4). Past infection with COVID-19 was also a significant predictor, increasing the odds of having used a telemedicine visit by 1.4.

### Gender and Access Barriers

Gender differences were observed for specific tools. While women were generally more likely to use telephone consultations and e-prescriptions, men reported higher use of the Internet Patient Account (OR 1.3, 95% CI 1.3‐1.3) and email consultations (OR 1.4, 95% CI 1.3‐1.4). Interestingly, the ability to arrange transportation was positively associated with telemedicine use; for example, those who could arrange transport with some difficulty were 1.3 times more likely to use a telemedicine visit than those who walked to their appointments (OR 1.3, 95% CI 1.3‐1.3).

## Discussion

### Support of Original Study Objectives

This study assessed the adoption of telemedicine in Poland and identified its key sociodemographic and health-related predictors in the postpandemic period, thereby addressing both of our original objectives. Our findings indicate that telemedicine use was not evenly distributed across the population and was more strongly associated with sociodemographic characteristics, particularly education level and place of residence, than with health care need alone. This pattern is consistent with the hypotheses formulated within the Digital Health Equity Framework: eHealth adoption in postpandemic Poland appears to be driven more by socioeconomic status than by clinical need, and telemedicine currently functions more as a tool of convenience for the already connected than as a tool of necessity for the isolated. The sections that follow compare these findings with the existing literature and discuss their interpretation, generalizability, limitations, and implications.

### Comparisons With Existing Literature

National-scale analysis of telemedicine adoption makes this study unlike many previous studies that focus on a limited number of services or small, specific populations. Our research provides a comprehensive view of telemedicine use across various sociodemographic and clinical factors, offering valuable insights into digital health disparities [12-15,27-30]. This study contributes by providing new evidence on the predictors of telemedicine adoption, particularly highlighting the digital divide related to education and urbanity. It offers critical data for policymakers, showing which groups are most at risk of being excluded from digital health care services and providing a clear direction for targeted interventions.

The only work worth mentioning, but not suitable for comparison, is an assessment of teleconsultation potential use from the perspective of health care providers and patients conducted in Poland by Lubomski et al [25]. The authors distributed an online questionnaire to 2318 patients and health care workers. They did not analyze the sociodemographic factors, but observed a higher attitude toward teleconsultation use among medical professionals and an interesting “conflict of interests”—each group (both the providers and the patients) declared that they should be the one to decide about the consultation form [25].

The most pronounced disparity observed in our study was the geographic and educational gap. This finding stands in stark contrast to the widely held promise that telemedicine can reduce inequalities by improving access for rural and underserved populations [31,32]. Our data show the opposite is occurring in Poland: residents of the largest cities consistently reported more frequent use of nearly all telemedicine services than those in small towns and rural areas. This pattern, where higher education and urban living predict greater adoption, is consistent with findings from other health care systems, such as in the United States, where Luo et al [17] found videoconference visits were more common among patients with private insurance and a college education. This suggests that digital exclusion is a pervasive challenge that technology alone cannot solve. Interestingly, our findings on age diverge from some international contexts; for example, a study in the United Arab Emirates found that older age was associated with higher telemedicine use [20], whereas our data point to younger, more digitally native cohorts being the primary users in Poland.

### Interpretation

This large-scale national study provides a comprehensive snapshot of telemedicine adoption in Poland’s postpandemic era, revealing a critical insight: rather than bridging the health care access gap, the digital transformation has largely mirrored, and in some cases may be reinforcing, existing socioeconomic divides. The research was conducted within the CHERRIES (Checklist for Reporting Results of Internet E-Surveys) methodology (Checklist 1). Our principal finding is that the most significant predictors of eHealth use are not clinical need alone, but are overwhelmingly driven by sociodemographic factors, principally higher education and urban residence. While the COVID-19 pandemic mandated a rapid expansion of remote care, with up to 80% of primary care appointments taking place remotely in its first year [1,24], our results indicate that the long-term patterns of adoption are creating a distinct digital divide between different segments of the Polish population.

Perhaps our most counterintuitive finding challenges the foundational promise of telemedicine as a solution for physical isolation. We found that individuals who could arrange transportation to a clinic—even with some difficulty—were more likely (OR 1.3, 95% CI 1.3‐1.3) to use telemedicine than those who walked to appointments. This strongly suggests that, for the majority of current users, telemedicine is not primarily a tool of necessity for the physically isolated, but rather a “tool of convenience” for the already connected. This dynamic risks creating a 2-tiered system where the well-resourced enjoy enhanced access through both physical and digital channels, while the isolated face compounded barriers.

Furthermore, our analysis sheds light on the nature of telemedicine use. The high adoption rates for passive, system-driven tools like e-prescriptions must be viewed in the context of their obligatory implementation in 2020 [3,4]. This high “forced” adoption stands in stark contrast to proactive, patient-driven tools like video consultations or health apps, which require higher digital literacy and remain largely unused. The current model excels at obligatory transactional tasks but falls short of fostering active patient engagement. While patients in our study, and in others, report high levels of satisfaction with these convenient services [27,33], this pattern raises questions about the quality and depth of digital care. The current model in Poland appears to excel at transactional tasks, such as those needed for chronic disease management, but may be falling short of fostering the active patient engagement necessary for comprehensive digital health management [28-30].

The dominance of higher education as a predictor likely operates through multiple mechanisms. It serves as a proxy not only for digital health literacy—the ability to find, evaluate, and apply electronic health information—but also for higher trust in digital systems and greater financial resources for modern devices. Similarly, the urban-rural gap is likely driven by persistent disparities in broadband infrastructure quality in Poland, making video consultations technologically unfeasible for many rural residents regardless of their willingness to adopt them [3].

### Generalizability

Regarding the generalizability of these findings, our results apply most directly to adult internet users in Poland, who constitute the large majority of the adult population given that 95.9% of Polish households had internet access in 2023 [6]. Within this population, several features of the study support broad applicability: the sample of more than one million respondents covers the full adult age range (18‐99 y), all categories of settlement size from rural areas to the largest cities, and all levels of education, and the direction of the observed associations was consistent across the 12 eHealth services examined. The sociodemographic gradients we identified also mirror those reported in nationally representative studies from other health care systems [16,18,21], which suggests that our conclusions about the social patterning of telemedicine use are not idiosyncratic to the Polish context and may be informative for other Central and Eastern European countries with similar postpandemic eHealth trajectories. At the same time, because participation required internet access and was self-selected through a health portal, our estimates should not be extrapolated to the offline segment of the population, in which digital exclusion is by definition complete; this boundary of generalizability is examined in detail below.

### Limitations

This study has several limitations that must be considered. First and most critically, the data are derived from a large, online, self-selected convenience sample from a health portal. While this yields massive statistical power, it inherently selects for a population that is already online, more health-conscious, and more digitally literate than the general Polish public. Crucially, this means our findings likely underestimate the true extent of the digital divide, as the most excluded individuals—those completely offline—are not represented here. The profound disparities we observed within this already-connected group suggest that the true population-level divide is likely far wider. Our results should therefore be interpreted as a conservative estimate, or the “tip of the iceberg,” of digital exclusion in Poland. Second, data were self-reported over a 12-month recall period, introducing potential recall bias. Third, the cross-sectional design precludes causal inference; we cannot definitively state that low education causes low adoption, only that they are strongly linked. Finally, we lacked direct measures of digital literacy or trust in health care systems, which are likely important unmeasured confounders.

### Implications

These findings carry significant policy implications. Efforts to improve telemedicine adoption that focus solely on technological factors like service quality or system usability are unlikely to be sufficient [29]. Our study clearly identifies the populations at risk of being left behind by the digital health revolution: older adults, individuals with lower educational attainment, and residents of rural areas. To prevent the digital divide from becoming a permanent health inequality chasm, targeted interventions are urgently needed. These must include robust, state-supported digital literacy programs for patients and investment in technological infrastructure in less urbanized regions. Without such a concerted effort, Poland’s otherwise successful eHealth implementation risks exacerbating, rather than mitigating, the very health disparities it has the potential to solve.

Our findings suggest that efforts to bridge the digital divide in telemedicine should focus not only on microlevel improvements such as usability or service quality but also on mesolevel interventions involving health systems, organizations, and communities. Targeted policy interventions should therefore address the key predictors of telemedicine adoption identified in this study. These predictors, including sociodemographic factors such as education level, place of residence, and age, as well as health status, barriers to access, and gender differences, highlight the need for a multifaceted policy approach. Research highlights the pivotal role of organizational factors, such as leadership, infrastructure, and training, in supporting telemedicine adoption [34]. Recognizing both the potential and limitations of this new technology is crucial for helping providers maintain the personalized care that patients expect and that fosters strong relationships [35,36]. Effective communication with patients is also essential in this process, as it ensures that they feel they can interact with their health care provider and fully comprehend the health information, just as they would in an in-office visit, reinforcing the case for ongoing telemedicine use in primary care [37,38]. Specifically, mesolevel actors, including health care organizations and community networks, can address barriers to access and literacy by facilitating staff training, providing infrastructure support, and ensuring integration into routine health care workflows [39]. Furthermore, institutional change and diffusion of digital innovations across health systems can be vital for overcoming digital literacy challenges, particularly in underserved populations [22]. These organizations can also support community-based initiatives, such as digital literacy programs, in collaboration with local stakeholders to foster trust and engagement, complementing state-sponsored macrolevel policies [40]. By embedding equity goals into health system strategies and fostering interorganizational learning networks, mesolevel interventions can significantly contribute to closing the digital divide in telemedicine. The findings from this study emphasize the need for targeted policy interventions, such as digital literacy programs for older adults and expanded broadband in rural areas, to ensure equitable access to telemedicine [22,39,40].

Our results also point to directions for future research. Longitudinal studies could track how patterns of telemedicine use evolve as the postpandemic system matures and could evaluate the impact of specific interventions, such as digital literacy programs or broadband expansion, on adoption among currently underserved groups. Cohort studies incorporating direct measures of digital health literacy, trust, and infrastructure quality would help clarify the mechanisms underlying the educational and geographic gradients observed here, and qualitative research among nonusers could identify modifiable barriers that large-scale surveys cannot capture.

### Conclusions

In the postpandemic era, the expansion of telemedicine in Poland has not yet succeeded in bridging the health care access gap. Our findings, drawn from a comprehensive national study of over one million adults, demonstrate that the adoption of eHealth is driven less by clinical need and more by pre-existing socioeconomic advantages, principally higher education and urban residence. This has resulted in a digital divide where telemedicine acts more as a tool of convenience for the already connected rather than a tool of necessity for the underserved. To prevent this digital divide from becoming a permanent chasm, we recommend a multilevel policy approach. At the patient level, Poland needs state-supported, community-based digital literacy programs targeted specifically at older adults and those with primary education, moving beyond standard online tutorials. At the infrastructure level, continued investment in rural broadband is essential to make ’active’ tools like video consultations feasible outside major cities. Critically, at the systemic level, policymakers must mandate that health care providers maintain robust in-person (hybrid) alternatives to prevent the complete exclusion of the digitally unconnected. Ensuring this powerful transformation promotes equity is now the defining public health challenge for Poland’s digital future.

## Supplementary material

10.2196/88442 Multimedia Appendix 1 Demographic characteristics and health status of the full surveyed sample in a nationwide cross-sectional online survey on telemedicine use in Poland, 2020‐2024 (N=1,196,102).

10.2196/88442 Multimedia Appendix 2 Factors influencing use of telemedicine. Logistic regression models among adult participants in a nationwide cross-sectional online survey conducted in Poland between 2020-2024.

10.2196/88442 Multimedia Appendix 3 Factors associated with telemedicine use: ordered logistic regression model fitted on the analytic sample (n=820,000), nationwide cross-sectional online survey, Poland, 2020‐2024.

10.2196/88442 Multimedia Appendix 4 Factors influencing on use of telemedicine. Ordered logistic regression model among adult participants in a nationwide cross-sectional online survey conducted in Poland between 2020-2024.

10.2196/88442 Checklist 1 CHERRIES checklist.

## Abbreviations

CHERRIES: Checklist for Reporting Results of Internet E-Surveys
JARS-QUANT: Journal Article Reporting Standards for Quantitative Research
OR: odds ratio
WHO: World Health Organization
