# Supplementary material for: Patterns of Telemedicine Use and the Digital Divide Among Adults in Poland’s Postpandemic Era: Nationwide Cross-Sectional Survey
Source: J Med Internet Res. 2026 Sep 22;28:e88442. doi: 10.2196/88442 (PMC13597080; doi:10.2196/88442)
Supplement: Supplementary file 1 [file jmir-v28-e88442-s005.pdf]

CHERRIES: Patterns of Telemedicine Use and the Digital Divide Among Adults in Poland's Post-Pandemic Era: A Nationwide Cross-Sectional Survey

Checklist for Reporting Results of Internet E-Surveys

Item Category	*Checklist Item*	*Yes/No*	*Comments*
**Design**			
	Describe survey design	**Yes**	The study used a cross-sectional design, targeting Polish adults via a convenience sample (online health portal).
**IRB (Institutional Review Board) approval and informed consent process**			
	IRB approval	**Yes**	The study protocol was reviewed by the Bioethics Committee of the Military Chamber of Physicians in Warsaw, Poland (decision no. KB 65/2024), which determined that formal ethical approval was not required.
	Informed consent	**Yes**	All participants provided informed consent electronically before beginning the questionnaire by accepting the survey terms and conditions. Participants were informed about the purpose of the survey, and participation was voluntary. The data were collected anonymously, and only deidentified data were used for research purposes.
	Data protection	**Yes**	No personal identifiable information was collected. The survey was conducted anonymously, and only fully anonymized data were used for analysis.
**Development and pre-testing**			

	Development and testing	**Yes**	The questionnaire was developed as part of the National Test for Poles' Health, a large-scale nationwide online survey conducted annually in Poland. The items assessing e-health service use were specifically designed for this survey and administered electronically via the Medonet platform. The usability and technical functionality of the electronic questionnaire were tested internally before fielding.
**Recruitment process and description of the sample having access to the questionnaire**			
	Open survey versus closed survey	**Yes**	This was an open survey distributed via the Medonet online platform. Participation was voluntary and open to all adult internet users who accessed the survey through advertisements and articles on the Medonet website and its associated channels.
	Contact mode	**Yes**	Initial contact with participants was made via the Internet through the Medonet online platform.
	Advertising the survey	**Yes**	The survey was advertised online through articles and advertisements on the Medonet health platform and its associated channels. Participation was voluntary and open to adult website users.
**Survey administration**			
	Web/E-mail	**Yes**	The survey was conducted as a Web-based questionnaire distributed via the Medonet online platform.
	Context	**Yes**	The survey was hosted on Medonet, a large Polish health-focused online platform providing evidence-based information on health, medicine, and healthy lifestyles. The website primarily attracts individuals interested in health-related topics
	Mandatory/ voluntary	**Yes**	Participation in the survey was voluntary.
	Incentives	**No**	Participation was voluntary, and no financial or other incentives were provided to participants.

	Time/Date	**Yes**	Data were collected between 2020 and 2024 as part of the National Test for Poles' Health.
	Randomization of items or questionnaires	**No**	No randomization was used.
	Adaptive questioning	**Yes**	Adaptive questioning was used, and certain items were displayed conditionally based on participants' previous responses.
	Number of Items	Yes	The exact number of items varied slightly due to adaptive questioning, but the core telemedicine and health status modules consisted of approximately not more than 60 items.
	Number of screens (pages)	Yes	The questionnaire was distributed over not more than 35 screens/pages.
	Completeness check	**Yes**	Completeness was technically enforced using system validations (e.g., JavaScript/HTML) that required participants to select a response before proceeding to the next page.
	Review step	**Yes**	Participants were able to review and change their responses before submitting the questionnaire.
**Response rates**			
	Unique site visitor	Yes	Unique visitors were identified using a combination of IP addresses and browser cookies.
	View rate (Ratio of unique survey visitors/unique site visitors)	No	Not applicable. Data on the total unique visitors to the Medonet portal who viewed the survey invitation but did not click it were not systematically tracked.

	Participation rate (Ratio of unique visitors who agreed to participate/unique first survey page visitors)	No	Not applicable. The exact ratio of unique visitors who viewed the first page versus those who agreed to participate was not recorded.
	Completion rate (Ratio of users who finished the survey/users who agreed to participate)	Yes	The completion rate for the specific telemedicine and health status modules among those who initiated the survey was 68.6% (820,000 completed out of 1,196,102 recruited)."
**Preventing multiple entries from the same individual**			
	Cookies used	Yes	Cookies were used to assign a unique identifier to each user's browser to prevent duplicate entries.
	IP check	Yes	The IP addresses of client computers were tracked to identify and prevent potential duplicate submissions from the same user.
	Log file analysis	No	Log file analysis was not utilized.
	Registration	No	Registration or user login was not required, as this was an open survey.
**Analysis**			
	Handling of incomplete questionnaires	**Yes**	Yes. Questionnaires with incomplete responses in the telemedicine module were excluded, leaving a final analytic sample of 820,000.

	Questionnaires submitted with an atypical timestamp	**No**	Questionnaires were not excluded based on completion time, and no time-based cut-off was applied.
	Statistical correction	**No**	No statistical weighting or propensity score adjustments were applied to account for the non-representative nature of the sample.
