# Supplementary material for: Patterns of Telemedicine Use and the Digital Divide Among Adults in Poland’s Postpandemic Era: Nationwide Cross-Sectional Survey
Source: J Med Internet Res. 2026 Sep 22;28:e88442. doi: 10.2196/88442 (PMC13597080; doi:10.2196/88442)
Supplement: Supplementary file 2 [file jmir-v28-e88442-s001.docx]

| Feature | Total | Women | Men | Women-men difference (P value; effect size) |
| --- | --- | --- | --- | --- |
| Gender, n (%) | 1,196,102 (100) | 700,408 (58.6) | 495,694 (41.4) | P<.001; φ=0.17 |
| Age (y) |  |  |  | P<.001; rg a=0.05 |
| Mean (SD) | 51.8 (14.9) | 51.0 (14.7) | 52.8 (15.2) |  |
| Median (IQR) | 52.0 (40.0‐64.0) | 52.0 (40.0‐63.0) | 53.0 (41.0‐65.0) |  |
| Min-max | 18.0‐99.0 | 18.0‐99.0 | 18.0‐99.0 |  |
| Age category (y), n (%) |  |  |  | P<.001; Vcb=0.04 |
| 18‐24 | 41,346 (3.5) | 28,264 (2.4)c | 13,082 (1.1) |  |
| 25‐34 | 131,846 (11) | 78,614 (6.6)c | 53,232 (4.5) |  |
| 35‐44 | 226,795 (19) | 133,471 (11.2)c | 93,324 (7.8) |  |
| 45‐54 | 248,076 (20.7) | 150,873 (12.6)c | 97,203 (8.1) |  |
| 55 | 548,039 (45.9) | 309,186 (25.9) | 238,853 (20)c |  |
| Place of residence, n (%) |  |  |  | P<.001; Vc=0.04 |
| Rural | 252,448 (21.1) | 154,318 (12.9)c | 98,130 (8.2) |  |
| City (up to 19,000 citizens) | 137,196 (11.5) | 79,651 (6.7) | 57,545 (4.8)c |  |
| City (20,000‐49,000 citizens) | 176,106 (14.7) | 107,289 (9)c | 68,817 (5.8) |  |
| City (50,000‐99,000 citizens) | 135,337 (11.3) | 80,014 (6.7)c | 55,323 (4.6) |  |
| City (100,000‐199,000 citizens) | 127,887 (10.7) | 73,897 (6.2) | 53,990 (4.5)c |  |
| City (200,000‐499,000 citizens) | 125,881 (10.5) | 74,201 (6.2)c | 51,680 (4.3) |  |
| City (500,000 or more citizens) | 241,247 (20.2) | 131,038 (11) | 110,209 (9.2)c |  |
| Education level, n (%) |  |  |  | P<.001; Vc=0.09 |
| Primary | 17,248 (1.4) | 11,011 (0.9)c | 6237 (0.5) |  |
| Lower secondary | 9890 (0.8) | 6430 (0.5)c | 3460 (0.3) |  |
| Basic vocational | 104,106 (8.7) | 50,954 (4.3) | 53,152 (4.4)c |  |
| Secondary | 339,438 (28.4) | 191,500 (16.0) | 147,938 (12.4)c |  |
| Postsecondary | 125,299 (10.5) | 90,628 (7.6)c | 34,671 (2.9) |  |
| Higher (bachelor or engineer) | 119,635 (10) | 61,999 (5.2) | 57,636 (4.8)c |  |
| Higher (master) | 480,486 (40.2) | 287,886 (24.1)c | 192,600 (16.1) |  |
| General physical health self-assessment, n (%) |  |  |  | P<.001; Vc=0.08 |
| Very good | 175,095 (14.6) | 90,522 (7.6) | 84,573 (7.1)c |  |
| Good | 504,212 (42.2) | 287,285 (24.0) | 216,927 (18.1)c |  |
| Average | 433,261 (36.2) | 269,806 (22.6)c | 163,455 (13.7) |  |
| Bad | 76,294 (6.4) | 48,302 (4.0)c | 27,992 (2.3) |  |
| Very bad | 7240 (0.6) | 4493 (0.4)c | 2747 (0.2) |  |
| General mental health self-assessment, n (%) |  |  |  | P<.001; Vc=0.12 |
| Very good | 285,949 (23.9) | 145,302 (12.1) | 140,647 (11.8) |  |
| Good | 511,182 (42.7) | 292,816 (24.5) | 218,366 (18.3) |  |
| Average | 294,111 (24.6) | 190,765 (15.9)c | 103,346 (8.6) |  |
| Bad | 92,763 (7.8) | 63,583 (5.3)c | 29,180 (2.4) |  |
| Very bad | 12,097 (1) | 7942 (0.7)c | 4155 (0.3) |  |
| Chronic and long-term diseases lasting 6 months or longer, n (%) |  |  |  | P<.001; φ=0.08 |
| Yes | 669,615 (56) | 416,576 (34.8)c | 253,039 (21.2) |  |
| No | 526,487 (44) | 283,832 (23.7) | 242,655 (20.3)c |  |
| Hypertension, n (%) |  |  |  | P<.001; φ=0.1 |
| Yes | 417,756 (34.9) | 215,913 (18.1) | 201,843 (16.9)c |  |
| No | 778,346 (65.1) | 484,495 (40.5)c | 293,851 (24.6) |  |
| Diabetes, n (%) |  |  |  | P<.001; φ=0.07 |
| Yes | 118,099 (9.9) | 57,051 (4.8) | 61,048 (5.1)c |  |
| No | 1,078,003 (90.1) | 643,357 (53.8)c | 434,646 (36.3) |  |
| Heart diseases, n (%) |  |  |  | P<.001; φ=0.03 |
| Yes | 159,208 (13.3) | 87,317 (7.3) | 71,891 (6.0)c |  |
| No | 1,036,894 (86.7) | 613,091 (51.3)c | 423,803 (35.4) |  |
| COPDd, n (%) |  |  |  | P<.001; φ=0.02 |
| Yes | 34,541 (2.9) | 18,676 (1.6) | 15,865 (1.3)c |  |
| No | 1,161,561 (97.1) | 681,732 (57)c | 479,829 (40.1) |  |
| Allergy or asthma, n (%) |  |  |  | P<.001; φ=0.08 |
| Yes | 239,007 (20) | 158,329 (13.2)c | 80,678 (6.8) |  |
| No | 957,095 (80) | 542,079 (45.3) | 415,016 (34.7)c |  |
| Cancer, n (%) |  |  |  | P<.001; φ=0.02 |
| Yes | 72,535 (6.1) | 45,463 (3.8)c | 27,072 (2.3) |  |
| No | 1,123,567 (93.9) | 654,945 (54.8) | 468,622 (39.9)c |  |
| Arthritis, n (%) |  |  |  | P<.001; φ=0.08 |
| Yes | 256,675 (21.5) | 169,455 (14.2)c | 87,220 (7.3) |  |
| No | 939,427 (78.5) | 530,953 (44.4) | 40,8474 (34.2)c |  |
| COVID-19, n (%) |  |  |  | P<.001; φ=0.04 |
| Yes | 237,880 (29) | 146,237 (17.8)c | 91,643 (11.2) |  |
| No | 582,120 (71) | 335,044 (40.9) | 247,076 (30.1)c |  |
| Depression diagnosis, n (%) |  |  |  | P<.001; φ=0.1 |
| Yes | 150,750 (12.6) | 107,854 (9.0)c | 42,896 (3.6) |  |
| No | 1,045,352 (87.4) | 592,554 (49.5) | 452,798 (37.9)c |  |
| Neurological disease, n (%) |  |  |  | P<.001; φ=0.05 |
| Yes | 133,985 (11.2) | 88,398 (7.4)c | 45,587 (3.8) |  |
| No | 1,062,117 (88.8) | 612,010 (51.2) | 450107 (37.6) S |  |
