# Supplementary material for: Patterns of Telemedicine Use and the Digital Divide Among Adults in Poland’s Postpandemic Era: Nationwide Cross-Sectional Survey
Source: J Med Internet Res. 2026 Sep 22;28:e88442. doi: 10.2196/88442 (PMC13597080; doi:10.2196/88442)
Supplement: Supplementary file 3 [file jmir-v28-e88442-s002.docx]

**Supplementary Data Table 1.** Factors influencing use of telemedicine. Logistic regression models among adult participants in a nationwide cross-sectional online survey conducted in Poland between 2020-2024..

| **Factors** | ***β*** | **OR** | **Lower 95 CI OR** | **Upper 95 CI OR** | ***P*** |
| --- | --- | --- | --- | --- | --- |
|  | | | | | |
| Model 1: Use of remote visit (online consultation. televising) via video conferencing system (0 = No. 1 = Yes) | | | | | |
|  | | | | | |
| Intercept | -3.11 | - | - | - | **-** |
| Age [years] | -.01 | 1.0 | 1.0 | 1.0 | **<.001** |
| Gender |  |  |  |  |  |
| female | reference |  |  |  |  |
| male | .04 | 1.0 | 1.0 | 1.1 | .076 |
| Education level |  |  |  |  |  |
| primary | .04 | 1.0 | 0.9 | 1.2 | .638 |
| lower secondary | -.21 | 0.8 | 0.6 | 1.1 | .198 |
| basic vocational | .01 | 1.0 | 0.9 | 1.1 | .765 |
| secondary | reference |  |  |  |  |
| post-secondary | .03 | 1.0 | 0.9 | 1.1 | .48 |
| higher (bachelor/engineer) | .18 | 1.2 | 1.1 | 1.3 | **<.001** |
| higher (master) | .15 | 1.2 | 1.1 | 1.2 | **<.001** |
| Place of residence |  |  |  |  | **<.001** |
| rural | -.17 | 0.8 | 0.8 | 0.9 | **<.001** |
| city up to 19k citizens | -.19 | 0.8 | 0.8 | 0.9 | **<.001** |
| city 20-49k citizens | -.02 | 1.0 | 0.9 | 1.1 | .596 |
| city 50-99k citizens | reference |  |  |  |  |
| city 100-199k citizens | -0.03 | 1.0 | 0.9 | 1.1 | .546 |
| city 200-499k citizens | .15 | 1.2 | 1.1 | 1.3 | **.002** |
| city 500k or more citizens | .44 | 1.6 | 1.4 | 1.7 | **<.001** |
| General physicall health self-assessment |  |  |  |  |  |
| very good | .11 | 1.1 | 1.0 | 1.2 | **.015** |
| good | .10 | 1.1 | 1.0 | 1.2 | **<.001** |
| average | reference |  |  |  |  |
| bad | -.04 | 1.0 | 0.9 | 1.0 | .365 |
| very bad | .08 | 1.1 | 0.9 | 1.3 | .415 |
| General mental health self-assessment |  |  |  |  |  |
| very good | -.002 | 1.0 | 0.9 | 1.1 | .964 |
| good | .04 | 1.0 | 1.0 | 1.1 | .174 |
| average | reference |  |  |  |  |
| bad | .08 | 1.1 | 1.0 | 1.2 | **.048** |
| very bad | .14 | 1.2 | 1.0 | 1.4 | .105 |
| Chronic and long-term diseases lasting 6 months or longer |  |  |  |  |  |
| yes | .18 | 1.2 | 1.1 | 1.3 | **<.001** |
| no | reference |  |  |  |  |
| Hypertension |  |  |  |  |  |
| yes | .07 | 1.1 | 1.0 | 1.1 | **.006** |
| no | reference |  |  |  |  |
| Diabetes |  |  |  |  |  |
| yes | .04 | 1.0 | 1.0 | 1.1 | .269 |
| no | reference |  |  |  |  |
| Heart diseases |  |  |  |  |  |
| yes | .08 | 1.1 | 1.0 | 1.2 | **.005** |
| no | reference |  |  |  |  |
| Chronic obstructive pulmonary disease (COPD) |  |  |  |  |  |
| yes | .09 | 1.1 | 1.0 | 1.2 | .096 |
| no | reference |  |  |  |  |
| Allergy or asthma |  |  |  |  |  |
| yes | .03 | 1.0 | 1.0 | 1.1 | .253 |
| no | reference |  |  |  |  |
| Depression diagnosis |  |  |  |  |  |
| yes | .38 | 1.5 | 1.4 | 1.5 | **<.001** |
| no | reference |  |  |  |  |
| Cancer |  |  |  |  |  |
| yes | .12 | 1.1 | 1.0 | 1.2 | **.001** |
| no | reference |  |  |  |  |
| Arthritis |  |  |  |  |  |
| yes | .04 | 1.0 | 1.0 | 1.1 | .181 |
| no | reference |  |  |  |  |
| Neurological disease |  |  |  |  |  |
| yes | .14 | 1.1 | 1.1 | 1.2 | **<.001** |
| no | reference |  |  |  |  |
| COVID-19 |  |  |  |  |  |
| yes | .14 | 1.2 | 1.1 | 1.2 | **<.001** |
| no | reference |  |  |  |  |
| Give up from financial reasons from a visit to the doctor |  |  |  |  |  |
| yes | .07 | 1.1 | 1.0 | 1.2 | .07 |
| no | reference |  |  |  |  |
| have never been put in this situation | -.18 | 0.8 | 0.8 | 0.9 | **<.001** |
| Give up from financial reasons from a dental visit |  |  |  |  |  |
| yes | .03 | 1.0 | 1.0 | 1.1 | .322 |
| no | reference |  |  |  |  |
| have never been put in this situation | .19 | 1.2 | 1.1 | 1.3 | **<.001** |
| Give up from financial reasons from purchasing a prescription drug |  |  |  |  |  |
| yes | .18 | 1.2 | 1.1 | 1.3 | **<.001** |
| no | reference |  |  |  |  |
| have never been put in this situation | -.07 | 0.9 | 0.9 | 1.0 | .091 |
|  |  |  |  |  |  |
| Give up from financial reasons from purchasing needed medical equipment |  |  |  |  |  |
| yes | .04 | 1.0 | 1.0 | 1.1 | .36 |
| no | reference |  |  |  |  |
| have never been put in this situation | -.25 | 0.8 | 0.7 | 0.8 | **<.001** |
| Opportunity to provide transportation to the medical appointment |  |  |  |  |  |
| yes. without any difficulty | .34 | 1.4 | 1.3 | 1.6 | **<.001** |
| yes. but with some difficulties | .45 | 1.6 | 1.4 | 1.8 | **<.001** |
| yes. but with great difficulties | .46 | 1.6 | 1.3 | 1.9 | **<.001** |
| not able to provide transportation to the place of medical appointment | reference |  |  |  |  |
| do not need transportation to the place of medical appointment - I walk there | .70 | 2.0 | 1.6 | 2.5 | **<.001** |
| difficult to say | .25 | 1.3 | 1.1 | 1.5 | **.008** |
|  |  |  |  |  |  |
|  | | | | | |
| Model 2: Use of remote visit (online consultation. televising) via telephone: logistic regression model among adult participants in a nationwide cross-sectional online survey conducted in Poland, 2020-2024. (0 = No. 1 = Yes) | | | | | |
|  | | | | | |
| Intercept | -1.11 | - | - | - | **-** |
| Age [years] | -.01 | 1.0 | 1.0 | 1.0 | **<.001** |
| Gender |  |  |  |  |  |
| female | reference |  |  |  |  |
| male | -.25 | 0.8 | 0.8 | 0.8 | **<.001** |
| Education level |  |  |  |  |  |
| primary | -.42 | 0.7 | 0.6 | 0.7 | **<.001** |
| lower secondary | -.36 | 0.7 | 0.6 | 0.8 | **<.001** |
| basic vocational | -.26 | 0.8 | 0.7 | 0.8 | **<.001** |
| secondary | reference |  |  |  |  |
| post-secondary | .07 | 1.1 | 1.0 | 1.1 | **.002** |
| higher (bachelor/engineer) | .24 | 1.3 | 1.2 | 1.3 | **<.001** |
| higher (master) | .34 | 1.4 | 1.4 | 1.4 | **<.001** |
| Place of residence |  |  |  |  |  |
| rural | .01 | 1.0 | 1.0 | 1.1 | .7845 |
| city up to 19k citizens | -.22 | 0.8 | 0.8 | 0.8 | **<.001** |
| city 20-49k citizens | -.04 | 1.0 | 0.9 | 1.0 | .12 |
| city 50-99k citizens | reference |  |  |  |  |
| city 100-199k citizens | .10 | 1.1 | 1.0 | 1.2 | **<.001** |
| city 200-499k citizens | .29 | 1.3 | 1.3 | 1.4 | **<.001** |
| city 500k or more citizens | .69 | 2.0 | 1.9 | 2.1 | **<.001** |
| General physicall health self-assessment |  |  |  |  |  |
| very good | -.07 | 0.9 | 0.9 | 1.0 | **.003** |
| good | .003 | 1.0 | 1.0 | 1.0 | .84 |
| average | reference |  |  |  |  |
| bad | .06 | 1.1 | 1.0 | 1.1 | **.006** |
| very bad | .16 | 1.2 | 1.0 | 1.3 | **.017** |
| General mental health self-assessment |  |  |  |  |  |
| very good | .02 | 1.0 | 1.0 | 1.1 | .298 |
| good | .05 | 1.0 | 1.0 | 1.1 | **.005** |
| average | reference |  |  |  |  |
| bad | -.06 | 0.9 | 0.9 | 1.0 | **.016** |
| very bad | -.21 | 0.8 | 0.7 | 0.9 | **<.001** |
| Chronic and long-term diseases lasting 6 months or longer |  |  |  |  |  |
| yes | .43 | 1.5 | 1.5 | 1.6 | **<.001** |
| no | reference |  |  |  |  |
| Hypertension |  |  |  |  |  |
| yes | .10 | 1.1 | 1.1 | 1.1 | **<.001** |
| no | reference |  |  |  |  |
| Diabetes |  |  |  |  |  |
| yes | .11 | 1.1 | 1.1 | 1.2 | **<.001** |
| no | reference |  |  |  |  |
| Heart diseases |  |  |  |  |  |
| yes | .08 | 1.1 | 1.0 | 1.1 | **<.001** |
| no | reference |  |  |  |  |
| Chronic obstructive pulmonary disease (COPD) |  |  |  |  |  |
| yes | .04 | 1.0 | 1.0 | 1.1 | .209 |
| no | reference |  |  |  |  |
| Allergy or asthma |  |  |  |  |  |
| yes | .15 | 1.2 | 1.1 | 1.2 | **<.001** |
| no | reference |  |  |  |  |
| Depression diagnosis |  |  |  |  |  |
| yes | .24 | 1.3 | 1.2 | 1.3 | **<.001** |
| no | reference |  |  |  |  |
| Cancer |  |  |  |  |  |
| yes | .19 | 1.2 | 1.2 | 1.3 | **<.001** |
| no | reference |  |  |  |  |
| Arthritis |  |  |  |  |  |
| yes | .03 | 1.0 | 1.0 | 1.1 | **.021** |
| no | reference |  |  |  |  |
| Neurological disease |  |  |  |  |  |
| yes | .18 | 1.2 | 1.2 | 1.2 | **<.001** |
| no | reference |  |  |  |  |
| COVID-19 |  |  |  |  |  |
| yes | .29 | 1.3 | 1.3 | 1.4 | **<.001** |
| no | reference |  |  |  |  |
| Give up from financial reasons from a visit to the doctor |  |  |  |  |  |
| yes | .12 | 1.1 | 1.1 | 1.2 | **<.001** |
| no | reference |  |  |  |  |
| have never been put in this situation | -.01 | 1.0 | 0.9 | 1.0 | .715 |
| Give up from financial reasons from a dental visit |  |  |  |  |  |
| yes | .12 | 1.1 | 1.1 | 1.2 | **<.001** |
| no | reference |  |  |  |  |
| have never been put in this situation | .09 | 1.1 | 1.0 | 1.1 | **<.001** |
| Give up from financial reasons from purchasing a prescription drug |  |  |  |  |  |
| yes | .17 | 1.2 | 1.1 | 1.2 | **<.001** |
| no | reference |  |  |  |  |
| have never been put in this situation | -.15 | 0.9 | 0.8 | 0.9 | **<.001** |
| Give up from financial reasons from purchasing needed medical equipment |  |  |  |  |  |
| yes | .12 | 1.1 | 1.1 | 1.2 | **<.001** |
| no | reference |  |  |  |  |
| have never been put in this situation | .02 | 1.0 | 1.0 | 1.1 | .257 |
| Opportunity to provide transportation to the medical appointment |  |  |  |  |  |
| yes. without any difficulty | .28 | 1.3 | 1.3 | 1.4 | **<.001** |
| yes. but with some difficulties | .40 | 1.5 | 1.4 | 1.6 | **<.001** |
| yes. but with great difficulties | .45 | 1.6 | 1.4 | 1.7 | **<.001** |
| not able to provide transportation to the place of medical appointment | reference |  |  |  |  |
| do not need transportation to the place of medical appointment - I walk there | .35 | 1.4 | 1.2 | 1.6 | **<.001** |
| difficult to say | .01 | 1.0 | 0.9 | 1.1 | .871 |
|  | | | | | |
| Model 3: E-prescriptions use: logistic regression model among adult participants in a nationwide cross-sectional online survey conducted in Poland, 2020-2024. (0 = No. 1 = Yes) | | | | | |
|  | | | | | |
| Intercept | -.16 | - | - | - | **-** |
| Age [years] | -.001 | 1.0 | 1.0 | 1.0 | **.032** |
| Gender |  |  |  |  |  |
| female | reference |  |  |  |  |
| male | -.11 | 0.9 | 0.9 | 0.9 | **<.001** |
| Education level |  |  |  |  |  |
| primary | -.61 | 0.5 | 0.5 | 0.6 | **<.001** |
| lower secondary | -.47 | 0.6 | 0.5 | 0.7 | **<.001** |
| basic vocational | -.46 | 0.6 | 0.6 | 0.7 | **<.001** |
| secondary | reference |  |  |  |  |
| post-secondary | .09 | 1.1 | 1.0 | 1.1 | **<.001** |
| higher (bachelor/engineer) | .36 | 1.4 | 1.4 | 1.5 | **<.001** |
| higher (master) | .51 | 1.7 | 1.6 | 1.7 | **<.001** |
| Place of residence |  |  |  |  |  |
| rural | -.03 | 1.0 | 0.9 | 1.0 | .173 |
| city up to 19k citizens | -.16 | 0.9 | 0.8 | 0.9 | **<.001** |
| city 20-49k citizens | -.05 | 1.0 | 0.9 | 1.0 | .059 |
| city 50-99k citizens | reference |  |  |  |  |
| city 100-199k citizens | .08 | 1.1 | 1.0 | 1.1 | **.005** |
| city 200-499k citizens | .16 | 1.2 | 1.1 | 1.2 | **<.001** |
| city 500k or more citizens | .32 | 1.4 | 1.3 | 1.5 | **<.001** |
| General physicall health self-assessment |  |  |  |  |  |
| very good | -.12 | 0.9 | 0.8 | 0.9 | **<.001** |
| good | .05 | 1.0 | 1.0 | 1.1 | **.006** |
| average | reference |  |  |  |  |
| bad | -.01 | 1.0 | 0.9 | 1.0 | .848 |
| very bad | -.06 | 0.9 | 0.8 | 1.1 | .423 |
| General mental health self-assessment |  |  |  |  |  |
| very good | .05 | 1.0 | 1.0 | 1.1 | **.049** |
| good | .04 | 1.0 | 1.0 | 1.1 | **.032** |
| average | reference |  |  |  |  |
| bad | -.02 | 1.0 | 0.9 | 1.0 | .524 |
| very bad | -.20 | 0.8 | 0.7 | 0.9 | **.002** |
| Chronic and long-term diseases lasting 6 months or longer |  |  |  |  |  |
| yes | .70 | 2.0 | 1.9 | 2.1 | **<.001** |
| no | reference |  |  |  |  |
| Hypertension |  |  |  |  |  |
| yes | .39 | 1.5 | 1.4 | 1.5 | **<.001** |
| no | reference |  |  |  |  |
| Diabetes |  |  |  |  |  |
| yes | .12 | 1.1 | 1.1 | 1.2 | **<.001** |
| no | reference |  |  |  |  |
| Heart diseases |  |  |  |  |  |
| yes | .10 | 1.1 | 1.1 | 1.1 | **<.001** |
| no | reference |  |  |  |  |
| Chronic obstructive pulmonary disease (COPD) |  |  |  |  |  |
| yes | -.05 | 1.0 | 0.9 | 1.0 | .144 |
| no | reference |  |  |  |  |
| Allergy or asthma |  |  |  |  |  |
| yes | .17 | 1.2 | 1.1 | 1.2 | **<.001** |
| no | reference |  |  |  |  |
| Depression diagnosis |  |  |  |  |  |
| yes | .14 | 1.1 | 1.1 | 1.2 | **<.001** |
| no | reference |  |  |  |  |
| Cancer |  |  |  |  |  |
| yes | .17 | 1.2 | 1.1 | 1.3 | **<.001** |
| no | reference |  |  |  |  |
| Arthritis |  |  |  |  |  |
| yes | .03 | 1.0 | 1.0 | 1.1 | **0.045** |
| no | reference |  |  |  |  |
| Neurological disease |  |  |  |  |  |
| yes | .11 | 1.1 | 1.1 | 1.2 | **<.001** |
| no | reference |  |  |  |  |
| COVID-19 |  |  |  |  |  |
| yes | .23 | 1.3 | 1.2 | 1.3 | **<.001** |
| no | reference |  |  |  |  |
| Give up from financial reasons from a visit to the doctor |  |  |  |  |  |
| yes | .06 | 1.1 | 1.0 | 1.1 | **.01** |
| no | reference |  |  |  |  |
| have never been put in this situation | -.06 | 0.9 | 0.9 | 1.0 | **0.01** |
| Give up from financial reasons from a dental visit |  |  |  |  |  |
| yes | .05 | 1.0 | 1.0 | 1.1 | **.023** |
| no | reference |  |  |  |  |
| have never been put in this situation | .08 | 1.1 | 1.0 | 1.1 | **<.001** |
| Give up from financial reasons from purchasing a prescription drug |  |  |  |  |  |
| yes | .04 | 1.0 | 1.0 | 1.1 | .083 |
| no | reference |  |  |  |  |
| have never been put in this situation | -.24 | 0.8 | 0.8 | 0.8 | **<.001** |
|  |  |  |  |  |  |
| Give up from financial reasons from purchasing needed medical equipment |  |  |  |  |  |
| yes | .03 | 1.0 | 1.0 | 1.1 | .243 |
| no | reference |  |  |  |  |
| have never been put in this situation | .03 | 1.0 | 1.0 | 1.1 | .122 |
| Opportunity to provide transportation to the medical appointment |  |  |  |  |  |
| yes. without any difficulty | .24 | 1.3 | 1.2 | 1.3 | **<.001** |
| yes. but with some difficulties | .19 | 1.2 | 1.1 | 1.3 | **<.001** |
| yes. but with great difficulties | .12 | 1.1 | 1.0 | 1.3 | **.048** |
| not able to provide transportation to the place of medical appointment | reference |  |  |  |  |
| do not need transportation to the place of medical appointment - I walk there | .05 | 1.0 | 0.9 | 1.2 | .525 |
| difficult to say | -.21 | 0.8 | 0.7 | 0.9 | **<.001** |
|  | | | | | |
| Model 4: E-referrals: logistic regression model among adult participants in a nationwide cross-sectional online survey conducted in Poland, 2020-2024. (0 = No. 1 = Yes) | | | | | |
|  | | | | | |
| Intercept | -.85 | - | - | - | **-** |
| Age [years] | -.002 | 1.0 | 1.0 | 1.0 | **<.001** |
| Gender |  |  |  |  |  |
| female | reference |  |  |  |  |
| male | -.06 | 0.9 | 0.9 | 1.0 | **<.001** |
| Education level |  |  |  |  |  |
| primary | -.54 | 0.6 | 0.5 | 0.6 | **<.001** |
| lower secondary | -.56 | 0.6 | 0.5 | 0.7 | **<.001** |
| basic vocational | -.43 | 0.6 | 0.6 | 0.7 | **<.001** |
| secondary | reference |  |  |  |  |
| post-secondary | .09 | 1.1 | 1.1 | 1.1 | **<.001** |
| higher (bachelor/engineer) | .30 | 1.4 | 1.3 | 1.4 | **<.001** |
| higher (master) | .49 | 1.6 | 1.6 | 1.7 | **<.001** |
| Place of residence |  |  |  |  |  |
| rural | -.06 | 0.9 | 0.9 | 1.0 | **.005** |
| city up to 19k citizens | -.14 | 0.9 | 0.8 | 0.9 | **<.001** |
| city 20-49k citizens | -.01 | 1.0 | 0.9 | 1.0 | .55 |
| city 50-99k citizens | reference |  |  |  |  |
| city 100-199k citizens | .11 | 1.1 | 1.1 | 1.2 | **<.001** |
| city 200-499k citizens | .24 | 1.3 | 1.2 | 1.3 | **<.001** |
| city 500k or more citizens | .32 | 1.4 | 1.3 | 1.4 | **<.001** |
| General physicall health self-assessment |  |  |  |  |  |
| very good | -.09 | 0.9 | 0.9 | 1.0 | **<.001** |
| good | .03 | 1.0 | 1.0 | 1.1 | .081 |
| average | reference |  |  |  |  |
| bad | -.01 | 1.0 | 0.9 | 1.0 | .767 |
| very bad | -.02 | 1.0 | 0.9 | 1.1 | .81 |
| General mental health self-assessment |  |  |  |  |  |
| very good | .01 | 1.0 | 1.0 | 1.0 | .735 |
| good | .03 | 1.0 | 1.0 | 1.1 | .073 |
| average | reference |  |  |  |  |
| bad | -.07 | 0.9 | 0.9 | 1.0 | .005 |
| very bad | -.19 | 0.8 | 0.7 | 0.9 | .001 |
| Chronic and long-term diseases lasting 6 months or longer |  |  |  |  |  |
| yes | .51 | 1.7 | 1.6 | 1.7 | **<.001** |
| no | reference |  |  |  |  |
| Hypertension |  |  |  |  |  |
| yes | .13 | 1.1 | 1.1 | 1.2 | **<.001** |
| no | reference |  |  |  |  |
| Diabetes |  |  |  |  |  |
| yes | .06 | 1.1 | 1.0 | 1.1 | **<.001** |
| no | reference |  |  |  |  |
| Heart diseases |  |  |  |  |  |
| yes | .09 | 1.1 | 1.1 | 1.1 | **<.001** |
| no | reference |  |  |  |  |
| Chronic obstructive pulmonary disease (COPD) |  |  |  |  |  |
| yes | .03 | 1.0 | 1.0 | 1.1 | .312 |
| no | reference |  |  |  |  |
| Allergy or asthma |  |  |  |  |  |
| yes | .11 | 1.1 | 1.1 | 1.1 | **<.001** |
| no | reference |  |  |  |  |
| Depression diagnosis |  |  |  |  |  |
| yes | -.01 | 1.0 | 1.0 | 1.0 | .699 |
| no | reference |  |  |  |  |
| Cancer |  |  |  |  |  |
| yes | .26 | 1.3 | 1.2 | 1.4 | **<.001** |
| no | reference |  |  |  |  |
| Arthritis |  |  |  |  |  |
| yes | .15 | 1.2 | 1.1 | 1.2 | **<.001** |
| no | reference |  |  |  |  |
| Neurological disease |  |  |  |  |  |
| yes | .28 | 1.3 | 1.3 | 1.4 | **<.001** |
| no | reference |  |  |  |  |
| COVID-19 |  |  |  |  |  |
| yes | .22 | 1.2 | 1.2 | 1.3 | **<.001** |
| no | reference |  |  |  |  |
| Give up from financial reasons from a visit to the doctor |  |  |  |  |  |
| yes | .07 | 1.1 | 1.0 | 1.1 | **<.001** |
| no | reference |  |  |  |  |
| have never been put in this situation | -.07 | 0.9 | 0.9 | 1.0 | **.002** |
| Give up from financial reasons from a dental visit |  |  |  |  |  |
| yes | .05 | 1.1 | 1.0 | 1.1 | **.008** |
| no | reference |  |  |  |  |
| have never been put in this situation | .07 | 1.1 | 1.0 | 1.1 | **.001** |
| Give up from financial reasons from purchasing a prescription drug |  |  |  |  |  |
| yes | .15 | 1.2 | 1.1 | 1.2 | **<.001** |
| no | reference |  |  |  |  |
| have never been put in this situation | -.13 | 0.9 | 0.8 | 0.9 | **<.001** |
|  |  |  |  |  |  |
| Give up from financial reasons from purchasing needed medical equipment |  |  |  |  |  |
| yes | .10 | 1.1 | 1.1 | 1.2 | **<.001** |
| no | reference |  |  |  |  |
| have never been put in this situation | -.02 | 1.0 | 0.9 | 1.0 | .144 |
| Opportunity to provide transportation to the medical appointment |  |  |  |  |  |
| yes. without any difficulty | .24 | 1.3 | 1.2 | 1.3 | **<.001** |
| yes. but with some difficulties | .23 | 1.3 | 1.2 | 1.3 | **<0.001** |
| yes. but with great difficulties | .08 | 1.1 | 1.0 | 1.2 | .126 |
| not able to provide transportation to the place of medical appointment | reference |  |  |  |  |
| do not need transportation to the place of medical appointment - I walk there | .04 | 1.0 | 0.9 | 1.2 | .525 |
| difficult to say | -.12 | 0.9 | 0.8 | 1.0 | **.009** |
|  | | | | | |
| Model 5: E-healthcare certificates: logistic regression model among adult participants in a nationwide cross-sectional online survey conducted in Poland, 2020-2024. (0 = No. 1 = Yes) | | | | | |
|  | | | | | |
| Intercept | .27 | - | - | - | **-** |
| Age [years] | -.05 | 0.9 | 0.9 | 1.0 | **<.001** |
| Gender |  |  |  |  |  |
| female | reference |  |  |  |  |
| male | .01 | 1.0 | 1.0 | 1.0 | .567 |
| Education level |  |  |  |  |  |
| primary | -.32 | 0.7 | 0.6 | 0.8 | **<.001** |
| lower secondary | -.60 | 0.6 | 0.4 | 0.7 | **<.001** |
| basic vocational | .07 | 1.1 | 1.0 | 1.1 | .052 |
| secondary | reference |  |  |  |  |
| post-secondary | .10 | 1.1 | 1.0 | 1.2 | **.003** |
| higher (bachelor/engineer) | .31 | 1.4 | 1.3 | 1.4 | **<.001** |
| higher (master) | .61 | 1.8 | 1.8 | 1.9 | **<.001** |
| Place of residence |  |  |  |  |  |
| rural | -.07 | 0.9 | 0.9 | 1.0 | **.03** |
| city up to 19k citizens | -.05 | 1.0 | 0.9 | 1.0 | .184 |
| city 20-49k citizens | -.01 | 1.0 | 0.9 | 1.1 | .841 |
| city 50-99k citizens | reference |  |  |  |  |
| city 100-199k citizens | -.04 | 1.0 | 0.9 | 1.0 | .255 |
| city 200-499k citizens | .03 | 1.0 | 1.0 | 1.1 | .438 |
| city 500k or more citizens | .11 | 1.1 | 1.1 | 1.2 | **<.001** |
| General physicall health self-assessment |  |  |  |  |  |
| very good | -.18 | 0.8 | 0.8 | 0.9 | **<.001** |
| good | -.06 | 0.9 | 0.9 | 1.0 | **.002** |
| average | reference |  |  |  |  |
| bad | .01 | 1.0 | 1.0 | 1.1 | .649 |
| very bad | -.03 | 1.0 | 0.8 | 1.1 | .727 |
| General mental health self-assessment |  |  |  |  |  |
| very good | -.07 | 0.9 | 0.9 | 1.0 | **.019** |
| good | -.02 | 1.0 | 0.9 | 1.0 | 0.322 |
| average | reference |  |  |  |  |
| bad | .002 | 1.0 | 0.9 | 1.0 | .322 |
| very bad | -.10 | 0.9 | 0.8 | 1.0 | .142 |
| Chronic and long-term diseases lasting 6 months or longer |  |  |  |  |  |
| yes | .23 | 1.3 | 1.2 | 1.3 | **<.001** |
| no | reference |  |  |  |  |
| Hypertension |  |  |  |  |  |
| yes | .05 | 1.0 | 1.0 | 1.1 | **.016** |
| no | reference |  |  |  |  |
| Diabetes |  |  |  |  |  |
| yes | -.09 | 0.9 | 0.9 | 1.0 | **.003** |
| no | reference |  |  |  |  |
| Heart diseases |  |  |  |  |  |
| yes | -.19 | 0.8 | 0.8 | 0.9 | **<.001** |
| no | reference |  |  |  |  |
| Chronic obstructive pulmonary disease (COPD) |  |  |  |  |  |
| yes | -.21 | 0.8 | 0.7 | 0.9 | **<.001** |
| no | reference |  |  |  |  |
| Allergy or asthma |  |  |  |  |  |
| yes | .04 | 1.0 | 1.0 | 1.1 | **.039** |
| no | reference |  |  |  |  |
| Depression diagnosis |  |  |  |  |  |
| yes | .14 | 1.2 | 1.1 | 1.2 | **<.001** |
| no | reference |  |  |  |  |
| Cancer |  |  |  |  |  |
| yes | .01 | 1.0 | 0.9 | 1.1 | .743 |
| no | reference |  |  |  |  |
| Arthritis |  |  |  |  |  |
| yes | -.04 | 1.0 | 0.9 | 1.0 | **.042** |
| no | reference |  |  |  |  |
| Neurological disease |  |  |  |  |  |
| yes | .08 | 1.1 | 1.0 | 1.1 | **<.001** |
| no | reference |  |  |  |  |
| COVID-19 |  |  |  |  |  |
| yes | .40 | 1.5 | 1.4 | 1.5 | **<.001** |
| no | reference |  |  |  |  |
| Give up from financial reasons from a visit to the doctor |  |  |  |  |  |
| yes | -.05 | 1.0 | 0.9 | 1.0 | .095 |
| no | reference |  |  |  |  |
| have never been put in this situation | .01 | 1.0 | 0.9 | 1.1 | .807 |
| Give up from financial reasons from a dental visit |  |  |  |  |  |
| yes | .20 | 1.2 | 1.2 | 1.3 | **<.001** |
| no | reference |  |  |  |  |
| have never been put in this situation | .05 | 1.1 | 1.0 | 1.1 | .104 |
| Give up from financial reasons from purchasing a prescription drug |  |  |  |  |  |
| yes | .19 | 1.2 | 1.1 | 1.3 | **<.001** |
| no | reference |  |  |  |  |
| have never been put in this situation | -.15 | 0.9 | 0.8 | 0.9 | **<.001** |
|  |  |  |  |  |  |
| Give up from financial reasons from purchasing needed medical equipment |  |  |  |  |  |
| yes | .13 | 1.1 | 1.1 | 1.2 | **<.001** |
| no | reference |  |  |  |  |
| have never been put in this situation | -.02 | 1.0 | 0.9 | 1.0 | .285 |
| Opportunity to provide transportation to the medical appointment |  |  |  |  |  |
| yes. without any difficulty | .26 | 1.3 | 1.2 | 1.4 | **<.001** |
| yes. but with some difficulties | .19 | 1.2 | 1.1 | 1.3 | **<.001** |
| yes. but with great difficulties | -.16 | 0.9 | 0.7 | 1.0 | .052 |
| not able to provide transportation to the place of medical appointment | reference |  |  |  |  |
| do not need transportation to the place of medical appointment - I walk there | -.09 | 0.9 | 0.7 | 1.1 | .377 |
| difficult to say | .02 | 1.0 | 0.9 | 1.2 | .796 |
|  | | | | | |
| Model 6: E-registrations use: logistic regression model among adult participants in a nationwide cross-sectional online survey conducted in Poland, 2020-2024.(0 = No. 1 = Yes) | | | | | |
|  | | | | | |
| Intercept | -2.21 | - | - | - | **-** |
| Age [years] | .01 | 1.0 | 1.0 | 1.0 | **<.001** |
| Gender |  |  |  |  |  |
| female | reference |  |  |  |  |
| male | .14 | 1.2 | 1.1 | 1.2 | **<.001** |
| Education level |  |  |  |  |  |
| primary | -.19 | 0.8 | 0.7 | 0.9 | **<.001** |
| lower secondary | -.26 | 0.8 | 0.7 | 0.9 | **.004** |
| basic vocational | -.13 | 0.9 | 0.8 | 0.9 | **<.001** |
| secondary | reference |  |  |  |  |
| post-secondary | .03 | 1.0 | 1.0 | 1.1 | .2 |
| higher (bachelor/engineer) | .19 | 1.2 | 1.2 | 1.3 | **<.001** |
| higher (master) | .27 | 1.3 | 1.3 | 1.3 | **<.001** |
| Place of residence |  |  |  |  |  |
| rural | .12 | 1.1 | 1.1 | 1.2 | **<.001** |
| city up to 19k citizens | -.06 | 0.9 | 0.9 | 1.0 | **.025** |
| city 20-49k citizens | -.02 | 1.0 | 0.9 | 1.0 | .461 |
| city 50-99k citizens | reference |  |  |  |  |
| city 100-199k citizens | .06 | 1.1 | 1.0 | 1.1 | **.016** |
| city 200-499k citizens | .30 | 1.4 | 1.3 | 1.4 | **<.001** |
| city 500k or more citizens | .52 | 1.7 | 1.6 | 1.8 | **<.001** |
| General physicall health self-assessment |  |  |  |  |  |
| very good | -.0001 | 1.0 | 1.0 | 1.0 | .997 |
| good | .04 | 1.0 | 1.0 | 1.1 | **.004** |
| average | reference |  |  |  |  |
| bad | .01 | 1.0 | 1.0 | 1.1 | .58 |
| very bad | .02 | 1.0 | 0.9 | 1.2 | 0.753 |
| General mental health self-assessment |  |  |  |  |  |
| very good | -.02 | 1.0 | 0.9 | 1.0 | .362 |
| good | -.001 | 1.0 | 1.0 | 1.0 | .955 |
| average | reference |  |  |  |  |
| bad | -.06 | 0.9 | 0.9 | 1.0 | **.02** |
| very bad | -.19 | 0.8 | 0.7 | 0.9 | **.001** |
| Chronic and long-term diseases lasting 6 months or longer |  |  |  |  |  |
| yes | .36 | 1.4 | 1.4 | 1.5 | **<.001** |
| no | reference |  |  |  |  |
| Hypertension |  |  |  |  |  |
| yes | .09 | 1.1 | 1.1 | 1.1 | **<.001** |
| no | reference |  |  |  |  |
| Diabetes |  |  |  |  |  |
| yes | .09 | 1.1 | 1.1 | 1.1 | **<.001** |
| no | reference |  |  |  |  |
| Heart diseases |  |  |  |  |  |
| yes | .13 | 1.1 | 1.1 | 1.2 | **<.001** |
| no | reference |  |  |  |  |
| Chronic obstructive pulmonary disease (COPD) |  |  |  |  |  |
| yes | .06 | 1.1 | 1.0 | 1.1 | .066 |
| no | reference |  |  |  |  |
| Allergy or asthma |  |  |  |  |  |
| yes | .05 | 1.0 | 1.0 | 1.1 | **<.001** |
| no | reference |  |  |  |  |
| Depression diagnosis |  |  |  |  |  |
| yes | .09 | 1.1 | 1.1 | 1.1 | **<.001** |
| no | reference |  |  |  |  |
| Cancer |  |  |  |  |  |
| yes | .20 | 1.2 | 1.2 | 1.3 | **<.001** |
| no | reference |  |  |  |  |
| Arthritis |  |  |  |  |  |
| yes | .11 | 1.1 | 1.1 | 1.2 | **<.001** |
| no | reference |  |  |  |  |
| Neurological disease |  |  |  |  |  |
| yes | .16 | 1.2 | 1.1 | 1.2 | **<.001** |
| no | reference |  |  |  |  |
| COVID-19 |  |  |  |  |  |
| yes | .17 | 1.2 | 1.2 | 1.2 | **<.001** |
| no | reference |  |  |  |  |
| Give up from financial reasons from a visit to the doctor |  |  |  |  |  |
| yes | .09 | 1.1 | 1.0 | 1.1 | **<.001** |
| no | reference |  |  |  |  |
| have never been put in this situation | -.11 | 0.9 | 0.9 | 0.9 | **<.001** |
| Give up from financial reasons from a dental visit |  |  |  |  |  |
| yes | .05 | 1.0 | 1.0 | 1.1 | **.015** |
| no | reference |  |  |  |  |
| have never been put in this situation | .09 | 1.1 | 1.0 | 1.1 | **<.001** |
| Give up from financial reasons from purchasing a prescription drug |  |  |  |  |  |
| yes | .08 | 1.1 | 1.0 | 1.1 | **<.001** |
| no | reference |  |  |  |  |
| have never been put in this situation | -.08 | 0.9 | 0.9 | 1.0 | **<.001** |
|  |  |  |  |  |  |
| Give up from financial reasons from purchasing needed medical equipment |  |  |  |  |  |
| yes | .13 | 1.1 | 1.1 | 1.2 | **<.001** |
| no | reference |  |  |  |  |
| have never been put in this situation | -.03 | 1.0 | 0.9 | 1.0 | .053 |
| Opportunity to provide transportation to the medical appointment |  |  |  |  |  |
| yes. without any difficulty | .30 | 1.3 | 1.3 | 1.4 | **<.001** |
| yes. but with some difficulties | .28 | 1.3 | 1.2 | 1.4 | **<.001** |
| yes. but with great difficulties | .20 | 1.2 | 1.1 | 1.4 | **<.001** |
| not able to provide transportation to the place of medical appointment | reference |  |  |  |  |
| do not need transportation to the place of medical appointment - I walk there | .03 | 1.0 | 0.9 | 1.2 | .696 |
| difficult to say | -.07 | 0.9 | 0.8 | 1.0 | .165 |
|  | | | | | |
| Model 7: Internet Patient Account: logistic regression model among adult participants in a nationwide cross-sectional online survey conducted in Poland, 2020-2024.(0 = No. 1 = Yes) | | | | | |
|  | | | | | |
| Intercept | .01 | - | - | - | **-** |
| Age [years] | -.02 | 1.0 | 1.0 | 1.0 | **<.001** |
| Gender |  |  |  |  |  |
| female | reference |  |  |  |  |
| male | .25 | 1.3 | 1.3 | 1.3 | **<.001** |
| Education level |  |  |  |  |  |
| primary | -.86 | 0.4 | 0.4 | 0.5 | **<.001** |
| lower secondary | -.73 | 0.5 | 0.4 | 0.6 | **<.001** |
| basic vocational | -.58 | 0.6 | 0.5 | 0.6 | **<.001** |
| secondary | reference |  |  |  |  |
| post-secondary | .18 | 1.2 | 1.1 | 1.2 | **<.001** |
| higher (bachelor/engineer) | .50 | 1.6 | 1.6 | 1.7 | **<.001** |
| higher (master) | .78 | 2.2 | 2.1 | 2.2 | **<.001** |
| Place of residence |  |  |  |  |  |
| rural | -.18 | 1.2 | 1.1 | 1.2 | **<.001** |
| city up to 19k citizens | -.16 | 1.6 | 1.6 | 1.7 | **<.001** |
| city 20-49k citizens | -.09 | 2.2 | 2.1 | 2.2 | **<.001** |
| city 50-99k citizens | reference |  |  |  |  |
| city 100-199k citizens | .05 | 1.1 | 1.0 | 1.1 | **.032** |
| city 200-499k citizens | .03 | 1.0 | 1.0 | 1.1 | .256 |
| city 500k or more citizens | .08 | 1.1 | 1.0 | 1.1 | **<.001** |
| General physicall health self-assessment |  |  |  |  |  |
| very good | -.02 | 1.0 | 0.9 | 1.0 | .396 |
| good | .05 | 1.1 | 1.0 | 1.1 | **<.001** |
| average | reference |  |  |  |  |
| bad | -.02 | 1.0 | 0.9 | 1.0 | .355 |
| very bad | -.02 | 1.0 | 0.9 | 1.1 | .729 |
| General mental health self-assessment |  |  |  |  |  |
| very good | .04 | 1.0 | 1.0 | 1.1 | .091 |
| good | .02 | 1.0 | 1.0 | 1.1 | .203 |
| average | reference |  |  |  |  |
| bad | .04 | 1.0 | 1.0 | 1.1 | .158 |
| very bad | -.04 | 1.0 | 0.9 | 1.1 | .446 |
| Chronic and long-term diseases lasting 6 months or longer |  |  |  |  |  |
| yes | .46 | 1.6 | 1.5 | 1.6 | **<.001** |
| no | reference |  |  |  |  |
| Hypertension |  |  |  |  |  |
| yes | .12 | 1.1 | 1.1 | 1.2 | **<.001** |
| no | reference |  |  |  |  |
| Diabetes |  |  |  |  |  |
| yes | .08 | 1.1 | 1.0 | 1.1 | **<.001** |
| no | reference |  |  |  |  |
| Heart diseases |  |  |  |  |  |
| yes | .01 | 1.0 | 1.0 | 1.0 | .509 |
| no | reference |  |  |  |  |
| Chronic obstructive pulmonary disease (COPD) |  |  |  |  |  |
| yes | -.07 | 0.9 | 0.9 | 1.0 | **.029** |
| no | reference |  |  |  |  |
| Allergy or asthma |  |  |  |  |  |
| yes | .10 | 1.1 | 1.1 | 1.1 | **<.001** |
| no | reference |  |  |  |  |
| Depression diagnosis |  |  |  |  |  |
| yes | .02 | 1.0 | 1.0 | 1.1 | .306 |
| no | reference |  |  |  |  |
| Cancer |  |  |  |  |  |
| yes | .12 | 1.1 | 1.1 | 1.2 | **<.001** |
| no | reference |  |  |  |  |
| Arthritis |  |  |  |  |  |
| yes | -.01 | 1.0 | 1.0 | 1.0 | .416 |
| no | reference |  |  |  |  |
| Neurological disease |  |  |  |  |  |
| yes | .07 | 1.1 | 1.0 | 1.1 | **<.001** |
| no | reference |  |  |  |  |
| COVID-19 |  |  |  |  |  |
| yes | .26 | 1.3 | 1.3 | 1.3 | **<.001** |
| no | reference |  |  |  |  |
| Give up from financial reasons from a visit to the doctor |  |  |  |  |  |
| yes | .04 | 1.0 | 1.0 | 1.1 | .093 |
| no | reference |  |  |  |  |
| have never been put in this situation | -.04 | 1.0 | 0.9 | 1.0 | .052 |
| Give up from financial reasons from a dental visit |  |  |  |  |  |
| yes | .07 | 1.1 | 1.0 | 1.1 | **<.001** |
| no | reference |  |  |  |  |
| have never been put in this situation | .03 | 1.0 | 1.0 | 1.1 | .239 |
| Give up from financial reasons from purchasing a prescription drug |  |  |  |  |  |
| yes | -.002 | 1.0 | 1.0 | 1.0 | .914 |
| no | reference |  |  |  |  |
| have never been put in this situation | -.07 | 0.9 | 0.9 | 1.0 | **<.001** |
|  |  |  |  |  |  |
| Give up from financial reasons from purchasing needed medical equipment |  |  |  |  |  |
| yes | -.02 | 1.0 | 0.9 | 1.0 | .466 |
| no | reference |  |  |  |  |
| have never been put in this situation | -.02 | 1.0 | 1.0 | 1.0 | .345 |
| Opportunity to provide transportation to the medical appointment |  |  |  |  |  |
| yes. without any difficulty | .3 | 1.4 | 1.3 | 1.4 | **<.001** |
| yes. but with some difficulties | .16 | 1.2 | 1.1 | 1.2 | **<.001** |
| yes. but with great difficulties | -.03 | 1.0 | 0.9 | 1.1 | .515 |
| not able to provide transportation to the place of medical appointment | reference |  |  |  |  |
| do not need transportation to the place of medical appointment - I walk there | -.16 | 0.9 | 0.7 | 1.0 | **.02** |
| difficult to say | -.17 | 0.8 | 0.8 | 0.9 | **<.001** |
|  | | | | | |
| Model 8: Use a website with test results. e.g.. imaging. laboratory results: logistic regression model among adult participants in a nationwide cross-sectional online survey conducted in Poland, 2020-2024. (0 = No. 1 = Yes) | | | | | |
|  | | | | | |
| Intercept | -.17 | - | - | - | **-** |
| Age [years] | -.02 | 1.0 | 1.0 | 1.0 | **<.001** |
| Gender |  |  |  |  |  |
| female | reference |  |  |  |  |
| male | -.11 | 0.9 | 0.9 | 0.9 | **<.001** |
| Education level |  |  |  |  |  |
| primary | -.85 | 0.4 | 0.4 | 0.5 | **<.001** |
| lower secondary | -.80 | 0.4 | 0.4 | 0.5 | **<.001** |
| basic vocational | -.54 | 0.6 | 0.6 | 0.6 | **<.001** |
| secondary | reference |  |  |  |  |
| post-secondary | .16 | 1.2 | 1.1 | 1.2 | **<.001** |
| higher (bachelor/engineer) | .41 | 1.5 | 1.4 | 1.6 | **<.001** |
| higher (master) | .71 | 2.0 | 2.0 | 2.1 | **<.001** |
| Place of residence |  |  |  |  |  |
| rural | .10 | 1.1 | 1.1 | 1.2 | **<.001** |
| city up to 19k citizens | -.07 | 0.9 | 0.9 | 1.0 | **.007** |
| city 20-49k citizens | .04 | 1.0 | 1.0 | 1.1 | .083 |
| city 50-99k citizens | reference |  |  |  |  |
| city 100-199k citizens | .14 | 1.2 | 1.1 | 1.2 | **<.001** |
| city 200-499k citizens | .29 | 1.3 | 1.3 | 1.4 | **<.001** |
| city 500k or more citizens | .44 | 1.6 | 1.5 | 1.6 | **<.001** |
| General physicall health self-assessment |  |  |  |  |  |
| very good | .04 | 1.0 | 1.0 | 1.1 | .062 |
| good | .07 | 1.1 | 1.0 | 1.1 | **<.001** |
| average | reference |  |  |  |  |
| bad | -.02 | 1.0 | 0.9 | 1.0 | .415 |
| very bad | -.03 | 1.0 | 0.9 | 1.1 | .683 |
| General mental health self-assessment |  |  |  |  |  |
| very good | .01 | 1.0 | 1.0 | 1.0 | .743 |
| good | .01 | 1.0 | 1.0 | 1.0 | .457 |
| average | reference |  |  |  |  |
| bad | -.05 | 1.0 | 0.9 | 1.0 | **.047** |
| very bad | -.22 | 0.8 | 0.7 | 0.9 | **<.001** |
| Chronic and long-term diseases lasting 6 months or longer |  |  |  |  |  |
| yes | .51 | 1.7 | 1.6 | 1.7 | **<.001** |
| no | reference |  |  |  |  |
| Hypertension |  |  |  |  |  |
| yes | .06 | 1.1 | 1.0 | 1.1 | **<.001** |
| no | reference |  |  |  |  |
| Diabetes |  |  |  |  |  |
| yes | .01 | 1.0 | 1.0 | 1.0 | .754 |
| no | reference |  |  |  |  |
| Heart diseases |  |  |  |  |  |
| yes | .05 | 1.1 | 1.0 | 1.1 | **.001** |
| no | reference |  |  |  |  |
| Chronic obstructive pulmonary disease (COPD) |  |  |  |  |  |
| yes | -.16 | 0.9 | 0.8 | 0.9 | **<.001** |
| no | reference |  |  |  |  |
| Allergy or asthma |  |  |  |  |  |
| yes | .09 | 1.1 | 1.1 | 1.1 | **<.001** |
| no | reference |  |  |  |  |
| Depression diagnosis |  |  |  |  |  |
| yes | .03 | 1.0 | 1.0 | 1.1 | .07 |
| no | reference |  |  |  |  |
| Cancer |  |  |  |  |  |
| yes | .23 | 1.3 | 1.2 | 1.3 | **<.001** |
| no | reference |  |  |  |  |
| Arthritis |  |  |  |  |  |
| yes | .02 | 1.0 | 1.0 | 1.0 | .263 |
| no | reference |  |  |  |  |
| Neurological disease |  |  |  |  |  |
| yes | .09 | 1.1 | 1.1 | 1.1 | **<.001** |
| no | reference |  |  |  |  |
| COVID-19 |  |  |  |  |  |
| yes | .31 | 1.4 | 1.3 | 1.4 | **<.001** |
| no | reference |  |  |  |  |
| Give up from financial reasons from a visit to the doctor |  |  |  |  |  |
| yes | .05 | 1.0 | 1.0 | 1.1 | **.03** |
| no | reference |  |  |  |  |
| have never been put in this situation | -.08 | 0.9 | 0.9 | 1.0 | **<.001** |
| Give up from financial reasons from a dental visit |  |  |  |  |  |
| yes | -.01 | 1.0 | 1.0 | 1.0 | .521 |
| no | reference |  |  |  |  |
| have never been put in this situation | -.02 | 1.0 | 0.9 | 1.0 | .273 |
| Give up from financial reasons from purchasing a prescription drug |  |  |  |  |  |
| yes | -.06 | 0.9 | 0.9 | 1.0 | **.01** |
| no | reference |  |  |  |  |
| have never been put in this situation | -.06 | 0.9 | 0.9 | 1.0 | **.009** |
|  |  |  |  |  |  |
| Give up from financial reasons from purchasing needed medical equipment |  |  |  |  |  |
| yes | .05 | 1.0 | 1.0 | 1.1 | .069 |
| no | reference |  |  |  |  |
| have never been put in this situation | .002 | 1.0 | 1.0 | 1.0 | .91 |
| Opportunity to provide transportation to the medical appointment |  |  |  |  |  |
| yes. without any difficulty | .36 | 1.4 | 1.4 | 1.5 | **<.001** |
| yes. but with some difficulties | .22 | 1.2 | 1.2 | 1.3 | **<.001** |
| yes. but with great difficulties | .09 | 1.1 | 1.0 | 1.2 | .09 |
| not able to provide transportation to the place of medical appointment | reference |  |  |  |  |
| do not need transportation to the place of medical appointment - I walk there | -.23 | 0.8 | 0.7 | 0.9 | **<.001** |
| difficult to say | -.16 | 0.9 | 0.8 | 0.9 | **.001** |
|  | | | | | |
| Model 9: Use a mobile application that supports a healthy lifestyle. such as activity: logistic regression model among adult participants in a nationwide cross-sectional online survey conducted in Poland, 2020-2024.(0 = No. 1 = Yes) | | | | | |
|  | | | | | |
| Intercept | -.58 | - | - | - | **-** |
| Age [years] | -.03 | 1.0 | 1.0 | 1.0 | **<.001** |
| Gender |  |  |  |  |  |
| female | reference |  |  |  |  |
| male | .22 | 1.2 | 1.2 | 1.3 | **<.001** |
| Education level |  |  |  |  |  |
| primary | -.44 | 0.6 | 0.6 | 0.7 | **<.001** |
| lower secondary | -.31 | 0.7 | 0.6 | 0.9 | **.007** |
| basic vocational | -.48 | 0.6 | 0.6 | 0.7 | **<.001** |
| secondary | reference |  |  |  |  |
| post-secondary | .20 | 1.2 | 1.2 | 1.3 | **<.001** |
| higher (bachelor/engineer) | .44 | 1.6 | 1.5 | 1.6 | **<.001** |
| higher (master) | .56 | 1.8 | 1.7 | 1.8 | **<.001** |
| Place of residence |  |  |  |  |  |
| rural | -.14 | 0.9 | 0.8 | 0.9 | **<.001** |
| city up to 19k citizens | -.09 | 0.9 | 0.9 | 1.0 | **.003** |
| city 20-49k citizens | -.06 | 0.9 | 0.9 | 1.0 | **.028** |
| city 50-99k citizens | reference |  |  |  |  |
| city 100-199k citizens | .05 | 1.1 | 1.0 | 1.1 | .094 |
| city 200-499k citizens | .09 | 1.1 | 1.0 | 1.2 | **.006** |
| city 500k or more citizens | .22 | 1.2 | 1.2 | 1.3 | **<.001** |
| General physicall health self-assessment |  |  |  |  |  |
| very good | .42 | 1.5 | 1.4 | 1.6 | **<.001** |
| good | .26 | 1.3 | 1.3 | 1.3 | **<.001** |
| average | reference |  |  |  |  |
| bad | -.12 | 0.9 | 0.8 | 0.9 | **<.001** |
| very bad | -.02 | 1.0 | 0.8 | 1.2 | .856 |
| General mental health self-assessment |  |  |  |  |  |
| very good | .03 | 1.0 | 1.0 | 1.1 | .248 |
| good | .04 | 1.0 | 1.0 | 1.1 | **.041** |
| average | reference |  |  |  |  |
| bad | -.12 | 0.9 | 0.8 | 0.9 | **<.001** |
| very bad | -.24 | 0.8 | 0.7 | 0.9 | **<.001** |
| Chronic and long-term diseases lasting 6 months or longer |  |  |  |  |  |
| yes | .19 | 1.2 | 1.2 | 1.3 | **<.001** |
| no | reference |  |  |  |  |
| Hypertension |  |  |  |  |  |
| yes | .09 | 1.1 | 1.1 | 1.1 | **<.001** |
| no | reference |  |  |  |  |
| Diabetes |  |  |  |  |  |
| yes | .01 | 1.0 | 1.0 | 1.1 | .793 |
| no | reference |  |  |  |  |
| Heart diseases |  |  |  |  |  |
| yes | .10 | 1.1 | 1.1 | 1.2 | **<.001** |
| no | reference |  |  |  |  |
| Chronic obstructive pulmonary disease (COPD) |  |  |  |  |  |
| yes | -.16 | 0.9 | 0.8 | 0.9 | **<.001** |
| no | reference |  |  |  |  |
| Allergy or asthma |  |  |  |  |  |
| yes | .12 | 1.1 | 1.1 | 1.2 | **<.001** |
| no | reference |  |  |  |  |
| Depression diagnosis |  |  |  |  |  |
| yes | .14 | 1.1 | 1.1 | 1.2 | **<.001** |
| no | reference |  |  |  |  |
| Cancer |  |  |  |  |  |
| yes | .02 | 1.0 | 1.0 | 1.1 | .419 |
| no | reference |  |  |  |  |
| Arthritis |  |  |  |  |  |
| yes | .02 | 1.0 | 1.0 | 1.1 | .418 |
| no | reference |  |  |  |  |
| Neurological disease |  |  |  |  |  |
| yes | -.01 | 1.0 | 0.9 | 1.0 | .755 |
| no | reference |  |  |  |  |
| COVID-19 |  |  |  |  |  |
| yes | .21 | 1.2 | 1.2 | 1.3 | **<.001** |
| no | reference |  |  |  |  |
| Give up from financial reasons from a visit to the doctor |  |  |  |  |  |
| yes | .03 | 1.0 | 1.0 | 1.1 | .278 |
| no | reference |  |  |  |  |
| have never been put in this situation | -.0004 | 1.0 | 0.9 | 1.1 | .989 |
| Give up from financial reasons from a dental visit |  |  |  |  |  |
| yes | .07 | 1.1 | 1.0 | 1.1 | **.002** |
| no | reference |  |  |  |  |
| have never been put in this situation | .02 | 1.0 | 1.0 | 1.1 | .447 |
| Give up from financial reasons from purchasing a prescription drug |  |  |  |  |  |
| yes | -.03 | 1.0 | 0.9 | 1.0 | .224 |
| no | reference |  |  |  |  |
| have never been put in this situation | -.09 | 0.9 | 0.9 | 1.0 | **.001** |
|  |  |  |  |  |  |
| Give up from financial reasons from purchasing needed medical equipment |  |  |  |  |  |
| yes | .07 | 1.1 | 1.0 | 1.1 | **.018** |
| no | reference |  |  |  |  |
| have never been put in this situation | -.12 | 0.9 | 0.9 | 0.9 | **<.001** |
| Opportunity to provide transportation to the medical appointment |  |  |  |  |  |
| yes. without any difficulty | .35 | 1.4 | 1.3 | 1.5 | **<.001** |
| yes. but with some difficulties | .22 | 1.2 | 1.1 | 1.4 | **<.001** |
| yes. but with great difficulties | .11 | 1.1 | 1.0 | 1.3 | .14 |
| not able to provide transportation to the place of medical appointment | reference |  |  |  |  |
| do not need transportation to the place of medical appointment - I walk there | -.05 | 0.9 | 0.8 | 1.2 | .607 |
| difficult to say | -.22 | 0.8 | 0.7 | 0.9 | **.004** |
|  | | | | | |
| Model 10: E-mail for advice from a doctor or other employee: logistic regression model among adult participants in a nationwide cross-sectional online survey conducted in Poland, 2020-2024. (0 = No. 1 = Yes) | | | | | |
|  | | | | | |
| Intercept | -3.10 | - | - | - | **-** |
| Age [years] | -.01 | 1.0 | 1.0 | 1.0 | **<.001** |
| Gender |  |  |  |  |  |
| female | reference |  |  |  |  |
| male | .32 | 1.4 | 1.3 | 1.4 | **<.001** |
| Education level |  |  |  |  |  |
| primary | -.36 | 0.7 | 0.6 | 0.8 | **<.001** |
| lower secondary | -.17 | 0.8 | 0.6 | 1.1 | .248 |
| basic vocational | -.24 | 0.8 | 0.7 | 0.9 | **<.001** |
| secondary | reference |  |  |  |  |
| post-secondary | .19 | 1.2 | 1.1 | 1.3 | **<.001** |
| higher (bachelor/engineer) | .34 | 1.4 | 1.3 | 1.5 | **<.001** |
| higher (master) | .43 | 1.5 | 1.5 | 1.6 | **<.001** |
| Place of residence |  |  |  |  |  |
| rural | .03 | 1.0 | 1.0 | 1.1 | .406 |
| city up to 19k citizens | -.10 | 0.9 | 0.8 | 1.0 | **.014** |
| city 20-49k citizens | -.02 | 1.0 | 0.9 | 1.1 | .54 |
| city 50-99k citizens | reference |  |  |  |  |
| city 100-199k citizens | .13 | 1.1 | 1.1 | 1.2 | **.001** |
| city 200-499k citizens | .29 | 1.3 | 1.2 | 1.4 | **<.001** |
| city 500k or more citizens | .48 | 1.6 | 1.5 | 1.7 | **<.001** |
| General physicall health self-assessment |  |  |  |  |  |
| very good | .20 | 1.2 | 1.1 | 1.3 | **<.001** |
| good | .13 | 1.1 | 1.1 | 1.2 | **<.001** |
| average | reference |  |  |  |  |
| bad | -.06 | 0.9 | 0.9 | 1.0 | .108 |
| very bad | .10 | 1.1 | 0.9 | 1.3 | .31 |
| General mental health self-assessment |  |  |  |  |  |
| very good | .13 | 1.1 | 1.1 | 1.2 | **<.001** |
| good | .09 | 1.1 | 1.0 | 1.1 | **<.001** |
| average | reference |  |  |  |  |
| bad | -.09 | 0.9 | 0.8 | 1.0 | **.017** |
| very bad | .01 | 1.0 | 0.9 | 1.2 | .928 |
| Chronic and long-term diseases lasting 6 months or longer |  |  |  |  |  |
| yes | .30 | 1.3 | 1.3 | 1.4 | **<.001** |
| no | reference |  |  |  |  |
| Hypertension |  |  |  |  |  |
| yes | .08 | 1.1 | 1.0 | 1.1 | **<.001** |
| no | reference |  |  |  |  |
| Diabetes |  |  |  |  |  |
| yes | .05 | 1.1 | 1.0 | 1.1 | .064 |
| no | reference |  |  |  |  |
| Heart diseases |  |  |  |  |  |
| yes | .08 | 1.1 | 1.0 | 1.1 | **.002** |
| no | reference |  |  |  |  |
| Chronic obstructive pulmonary disease (COPD) |  |  |  |  |  |
| yes | -.04 | 1.0 | 0.9 | 1.1 | .466 |
| no | reference |  |  |  |  |
| Allergy or asthma |  |  |  |  |  |
| yes | .05 | 1.1 | 1.0 | 1.1 | **.019** |
| no | reference |  |  |  |  |
| Depression diagnosis |  |  |  |  |  |
| yes | .19 | 1.2 | 1.1 | 1.3 | **<.001** |
| no | reference |  |  |  |  |
| Cancer |  |  |  |  |  |
| yes | .21 | 1.2 | 1.2 | 1.3 | **<.001** |
| no | reference |  |  |  |  |
| Arthritis |  |  |  |  |  |
| yes | .07 | 1.1 | 1.0 | 1.1 | **.003** |
| no | reference |  |  |  |  |
| Neurological disease |  |  |  |  |  |
| yes | .15 | 1.2 | 1.1 | 1.2 | **<.001** |
| no | reference |  |  |  |  |
| COVID-19 |  |  |  |  |  |
| yes | .15 | 1.2 | 1.1 | 1.2 | **<.001** |
| no | reference |  |  |  |  |
| Give up from financial reasons from a visit to the doctor |  |  |  |  |  |
| yes | .07 | 1.1 | 1.0 | 1.1 | **.025** |
| no | reference |  |  |  |  |
| have never been put in this situation | -.10 | 0.9 | 0.8 | 1.0 | **.009** |
| Give up from financial reasons from a dental visit |  |  |  |  |  |
| yes | -.01 | 1.0 | 0.9 | 1.1 | .818 |
| no | reference |  |  |  |  |
| have never been put in this situation | .05 | 1.0 | 1.0 | 1.1 | .192 |
| Give up from financial reasons from purchasing a prescription drug |  |  |  |  |  |
| yes | .07 | 1.1 | 1.0 | 1.1 | **.038** |
| no | reference |  |  |  |  |
| have never been put in this situation | .01 | 1.0 | 0.9 | 1.1 | .668 |
|  |  |  |  |  |  |
| Give up from financial reasons from purchasing needed medical equipment |  |  |  |  |  |
| yes | .18 | 1.2 | 1.1 | 1.3 | **<.001** |
| no | reference |  |  |  |  |
| have never been put in this situation | -.25 | 0.8 | 0.7 | 0.8 | **<.001** |
| Opportunity to provide transportation to the medical appointment |  |  |  |  |  |
| yes. without any difficulty | .52 | 1.7 | 1.5 | 1.8 | **<.001** |
| yes. but with some difficulties | .46 | 1.6 | 1.4 | 1.8 | **<.001** |
| yes. but with great difficulties | .49 | 1.6 | 1.4 | 1.9 | **<.001** |
| not able to provide transportation to the place of medical appointment | reference |  |  |  |  |
| do not need transportation to the place of medical appointment - I walk there | .50 | 1.6 | 1.3 | 2.0 | **<.001** |
| difficult to say | .17 | 1.2 | 1.0 | 1.4 | .056 |
|  | | | | | |
| Model 11: Portal/website offering paid health advice: logistic regression model among adult participants in a nationwide cross-sectional online survey conducted in Poland, 2020-2024. (0 = No. 1 = Yes) | | | | | |
|  | | | | | |
| Intercept | -2.75 | - | - | - | **-** |
| Age [years] | -.01 | 1.0 | 1.0 | 1.0 | **<.001** |
| Gender |  |  |  |  |  |
| female | reference |  |  |  |  |
| male | .26 | 1.3 | 1.2 | 1.4 | **<.001** |
| Education level |  |  |  |  |  |
| primary | -.29 | 0.7 | 0.6 | 0.9 | **.007** |
| lower secondary | -.23 | 0.8 | 0.6 | 1.1 | .188 |
| basic vocational | -.36 | 0.7 | 0.6 | 0.8 | **<.001** |
| secondary | reference |  |  |  |  |
| post-secondary | .21 | 1.2 | 1.1 | 1.3 | **<.001** |
| higher (bachelor/engineer) | .41 | 1.5 | 1.4 | 1.6 | **<.001** |
| higher (master) | .50 | 1.6 | 1.6 | 1.7 | **<.001** |
| Place of residence |  |  |  |  |  |
| rural | .07 | 1.1 | 1.0 | 1.2 | .082 |
| city up to 19k citizens | -.06 | 0.9 | 0.9 | 1.0 | .249 |
| city 20-49k citizens | -.02 | 1.0 | 0.9 | 1.1 | .734 |
| city 50-99k citizens | reference |  |  |  |  |
| city 100-199k citizens | .09 | 1.1 | 1.0 | 1.2 | **.047** |
| city 200-499k citizens | .31 | 1.4 | 1.2 | 1.5 | **<.001** |
| city 500k or more citizens | .55 | 1.7 | 1.6 | 1.9 | **<.001** |
| General physicall health self-assessment |  |  |  |  |  |
| very good | .18 | 1.2 | 1.1 | 1.3 | **<.001** |
| good | .12 | 1.1 | 1.1 | 1.2 | **<.001** |
| average | reference |  |  |  |  |
| bad | -.07 | 0.9 | 0.9 | 1.0 | .088 |
| very bad | .10 | 1.1 | 0.9 | 1.4 | .376 |
| General mental health self-assessment |  |  |  |  |  |
| very good | -.01 | 1.0 | 0.9 | 1.1 | .746 |
| good | .03 | 1.0 | 1.0 | 1.1 | .358 |
| average | reference |  |  |  |  |
| bad | -.003 | 1.0 | 0.9 | 1.1 | .935 |
| very bad | .005 | 1.0 | 0.8 | 1.2 | .959 |
| Chronic and long-term diseases lasting 6 months or longer |  |  |  |  |  |
| yes | .11 | 1.1 | 1.1 | 1.2 | **<.001** |
| no | reference |  |  |  |  |
| Hypertension |  |  |  |  |  |
| yes | .05 | 1.0 | 1.0 | 1.1 | .061 |
| no | reference |  |  |  |  |
| Diabetes |  |  |  |  |  |
| yes | -.06 | 0.9 | 0.9 | 1.0 | .114 |
| no | reference |  |  |  |  |
| Heart diseases |  |  |  |  |  |
| yes | .05 | 1.1 | 1.0 | 1.1 | .072 |
| no | reference |  |  |  |  |
| Chronic obstructive pulmonary disease (COPD) |  |  |  |  |  |
| yes | -.05 | 1.0 | 0.8 | 1.1 | .419 |
| no | reference |  |  |  |  |
| Allergy or asthma |  |  |  |  |  |
| yes | .06 | 1.1 | 1.0 | 1.1 | **.024** |
| no | reference |  |  |  |  |
| Depression diagnosis |  |  |  |  |  |
| yes | .19 | 1.2 | 1.1 | 1.3 | **<.001** |
| no | reference |  |  |  |  |
| Cancer |  |  |  |  |  |
| yes | .16 | 1.2 | 1.1 | 1.3 | **<.001** |
| no | reference |  |  |  |  |
| Arthritis |  |  |  |  |  |
| yes | .03 | 1.0 | 1.0 | 1.1 | .2 |
| no | reference |  |  |  |  |
| Neurological disease |  |  |  |  |  |
| yes | .05 | 1.1 | 1.0 | 1.1 | .088 |
| no | reference |  |  |  |  |
| COVID-19 |  |  |  |  |  |
| yes | .19 | 1.2 | 1.2 | 1.3 | **<.001** |

| no | reference |  |  |  |  |
| --- | --- | --- | --- | --- | --- |
| Give up from financial reasons from a visit to the doctor |  |  |  |  |  |
| yes | .16 | 1.2 | 1.1 | 1.3 | **<.001** |
| no | reference |  |  |  |  |
| have never been put in this situation | -.11 | 0.9 | 0.8 | 1.0 | **.008** |
| Give up from financial reasons from a dental visit |  |  |  |  |  |
| yes | -.001 | 1.0 | 0.9 | 1.1 | .965 |
| no | reference |  |  |  |  |
| have never been put in this situation | .06 | 1.1 | 1.0 | 1.1 | .174 |
| Give up from financial reasons from purchasing a prescription drug |  |  |  |  |  |
| yes | -.02 | 1.0 | 0.9 | 1.1 | .566 |
| no | reference |  |  |  |  |
| have never been put in this situation | -.01 | 1.0 | 0.9 | 1.1 | .749 |
|  |  |  |  |  |  |
| Give up from financial reasons from purchasing needed medical equipment |  |  |  |  |  |
| yes | .15 | 1.2 | 1.1 | 1.3 | **<.001** |
| no | reference |  |  |  |  |
| have never been put in this situation | -.24 | 0.8 | 0.7 | 0.8 | **<.001** |
| Opportunity to provide transportation to the medical appointment |  |  |  |  |  |
| yes. without any difficulty | .36 | 1.4 | 1.3 | 1.6 | **<.001** |
| yes. but with some difficulties | .38 | 1.5 | 1.3 | 1.6 | **<.001** |
| yes. but with great difficulties | .39 | 1.5 | 1.2 | 1.8 | **<.001** |
| not able to provide transportation to the place of medical appointment | reference |  |  |  |  |
| do not need transportation to the place of medical appointment - I walk there | .35 | 1.4 | 1.1 | 1.8 | **.006** |
| difficult to say | .02 | 1.0 | 0.8 | 1.3 | .857 |
|  | | | | | |
| Model 12: Use a portal/website offering the possibility to issue a prescription: logistic regression model among adult participants in a nationwide cross-sectional online survey conducted in Poland, 2020-2024. (0 = No. 1 = Yes) | | | | | |
|  | | | | | |
| Intercept | -2.94 | - | - | - | **-** |
| Age [years] | -.01 | 1.0 | 1.0 | 1.0 | **<.001** |
| Gender |  |  |  |  |  |
| female | reference |  |  |  |  |
| male | .17 | 1.2 | 1.1 | 1.2 | **<.001** |
| Education level |  |  |  |  |  |
| primary | -.10 | 0.9 | 0.7 | 1.1 | .301 |
| lower secondary | -.15 | 0.9 | 0.6 | 1.2 | .38 |
| basic vocational | -.17 | 0.8 | 0.8 | 0.9 | **<.001** |
| secondary | reference |  |  |  |  |
| post-secondary | .03 | 1.0 | 0.9 | 1.1 | .572 |
| higher (bachelor/engineer) | .31 | 1.4 | 1.2 | 1.5 | **<.001** |
| higher (master) | .24 | 1.3 | 1.2 | 1.3 | **<.001** |
| Place of residence |  |  |  |  |  |
| rural | -.05 | 1.0 | 0.9 | 1.0 | .284 |
| city up to 19k citizens | -.13 | 0.9 | 0.8 | 1.0 | **.016** |
| city 20-49k citizens | -.01 | 1.0 | 0.9 | 1.1 | .791 |
| city 50-99k citizens | reference |  |  |  |  |
| city 100-199k citizens | .06 | 1.1 | 1.0 | 1.2 | .221 |
| city 200-499k citizens | .18 | 1.2 | 1.1 | 1.3 | **<.001** |
| city 500k or more citizens | .47 | 1.6 | 1.5 | 1.8 | **<.001** |
| General physicall health self-assessment |  |  |  |  |  |
| very good | .12 | 1.1 | 1.0 | 1.2 | **.006** |
| good | .11 | 1.1 | 1.1 | 1.2 | **<.001** |
| average | reference |  |  |  |  |
| bad | -.09 | 0.9 | 0.8 | 1.0 | **.043** |
| very bad | -.11 | 0.9 | 0.7 | 1.1 | .371 |
| General mental health self-assessment |  |  |  |  |  |
| very good | -.01 | 1.0 | 0.9 | 1.1 | .745 |
| good | .04 | 1.0 | 1.0 | 1.1 | .167 |
| average | reference |  |  |  |  |
| bad | .05 | 1.1 | 1.0 | 1.1 | .21 |
| very bad | .24 | 1.3 | 1.1 | 1.5 | **.007** |
| Chronic and long-term diseases lasting 6 months or longer |  |  |  |  |  |
| yes | .15 | 1.2 | 1.1 | 1.2 | **<.001** |
| no | reference |  |  |  |  |
| Hypertension |  |  |  |  |  |
| yes | .18 | 1.2 | 1.1 | 1.3 | **<.001** |
| no | reference |  |  |  |  |
| Diabetes |  |  |  |  |  |
| yes | .07 | 1.1 | 1.0 | 1.1 | .055 |
| no | reference |  |  |  |  |
| Heart diseases |  |  |  |  |  |
| yes | .12 | 1.1 | 1.1 | 1.2 | **<.001** |
| no | reference |  |  |  |  |
| Chronic obstructive pulmonary disease (COPD) |  |  |  |  |  |
| yes | -.05 | 1.0 | 0.8 | 1.1 | .427 |
| no | reference |  |  |  |  |
| Allergy or asthma |  |  |  |  |  |
| yes | -.02 | 1.0 | 0.9 | 1.0 | .363 |
| no | reference |  |  |  |  |
| Depression diagnosis |  |  |  |  |  |
| yes | .31 | 1.4 | 1.3 | 1.4 | **<.001** |
| no | reference |  |  |  |  |
| Cancer |  |  |  |  |  |
| yes | .09 | 1.1 | 1.0 | 1.2 | **.031** |
| no | reference |  |  |  |  |
| Arthritis |  |  |  |  |  |
| yes | .001 | 1.0 | 0.9 | 1.1 | .966 |
| no | reference |  |  |  |  |
| Neurological disease |  |  |  |  |  |
| yes | -.06 | 0.9 | 0.9 | 1.0 | .099 |
| no | reference |  |  |  |  |
| COVID-19 |  |  |  |  |  |
| yes | .05 | 1.0 | 1.0 | 1.1 | .058 |
| no | reference |  |  |  |  |
| Give up from financial reasons from a visit to the doctor |  |  |  |  |  |
| yes | .17 | 1.2 | 1.1 | 1.3 | **<.001** |
| no | reference |  |  |  |  |
| have never been put in this situation | -.01 | 1.0 | 0.9 | 1.1 | .889 |
| Give up from financial reasons from a dental visit |  |  |  |  |  |
| yes | .07 | 1.1 | 1.0 | 1.2 | **.044** |
| no | reference |  |  |  |  |
| have never been put in this situation | .07 | 1.1 | 1.0 | 1.2 | .12 |
| Give up from financial reasons from purchasing a prescription drug |  |  |  |  |  |
| yes | .10 | 1.1 | 1.0 | 1.2 | **.015** |
| no | reference |  |  |  |  |
| have never been put in this situation | -.11 | 0.9 | 0.8 | 1.0 | **.008** |
|  |  |  |  |  |  |
| Give up from financial reasons from purchasing needed medical equipment |  |  |  |  |  |
| yes | .10 | 1.1 | 1.0 | 1.2 | **.022** |
| no | reference |  |  |  |  |
| have never been put in this situation | -.21 | 0.8 | 0.8 | 0.9 | **<.001** |
| Opportunity to provide transportation to the medical appointment |  |  |  |  |  |
| yes. without any difficulty | .56 | 1.8 | 1.6 | 2.0 | **<.001** |
| yes. but with some difficulties | .42 | 1.5 | 1.3 | 1.7 | **<.001** |
| yes. but with great difficulties | .50 | 1.7 | 1.3 | 2.0 | **<.001** |
| not able to provide transportation to the place of medical appointment | reference |  |  |  |  |
| do not need transportation to the place of medical appointment - I walk there | .53 | 1.7 | 1.3 | 2.2 | **<.001** |
| difficult to say | .12 | 1.1 | 0.9 | 1.4 | .316 |
