# Supplementary material for: Patterns of Telemedicine Use and the Digital Divide Among Adults in Poland’s Postpandemic Era: Nationwide Cross-Sectional Survey
Source: J Med Internet Res. 2026 Sep 22;28:e88442. doi: 10.2196/88442 (PMC13597080; doi:10.2196/88442)
Supplement: Supplementary file 4 [file jmir-v28-e88442-s003.docx]

| Factors | β | ORa (95% CI) | P value |
| --- | --- | --- | --- |
| Use of a telemedicine visit in the past 12 months (1=“no,” 2=“at least once,” and 3=“many times”) |  |  |  |
| Intercept 1 | −2.07 | 0.1 (0.1-0.1) | <.001 |
| Intercept 2 | −.44 | 0.6 (0.6-0.7) | <.001 |
| Age (y) | −.01 | 1.0 (1.0-1.0) | <.001 |
| Gender |  |  |  |
| Women | Reference |  |  |
| Men | −.37 | 0.7 (0.7-0.7) | <.001 |
| Education level |  |  |  |
| Primary | −.42 | 0.7 (0.6-0.7) | <.001 |
| Lower secondary | −.53 | 0.6 (0.6-0.6) | <.001 |
| Basic vocational | −.21 | 0.8 (0.8-0.8) | <.001 |
| Secondary | Reference |  |  |
| Postsecondary | .02 | 1.0 (1.0-1.0) | .0083 |
| Higher (bachelor or engineer) | .15 | 1.2 (1.1-1.2) | <.001 |
| Higher (master) | .23 | 1.3 (1.2-1.3) | <.001 |
| Place of residence |  |  |  |
| Rural | −.06 | 0.9 (0.9-1.0) | <.001 |
| City (up to 19,000 citizens) | −.19 | 0.8 (0.8-0.8) | <.001 |
| City (20,000‐49,000 citizens) | −.05 | 1.0 (0.9-1.0) | <.001 |
| City (50,000‐99,000 citizens) | Reference |  |  |
| City (100,000‐199,000 citizens) | .07 | 1.1 (1.1-1.1) | <.001 |
| City (200,000‐499,000 citizens) | .17 | 1.2 (1.2-1.2) | <.001 |
| City (500,000 or more citizens) | .43 | 1.5 (1.5-1.6) | <.001 |
| General physical health self-assessment |  |  |  |
| Very good | −.23 | 0.8 (0.8-0.8) | <.001 |
| Good | −.07 | 0.9 (0.9-0.9) | <.001 |
| Average | Reference |  |  |
| Bad | .09 | 1.1 (1.1-1.1) | <.001 |
| Very bad | .12 | 1.1 (1.1-1.2) | <.001 |
| General mental health self-assessment |  |  |  |
| Very good | −.03 | 1.0 (1.0-1.0) | <.001 |
| Good | .01 | 1.0 (1.0-1.0) | .019 |
| Average | Reference |  |  |
| Bad | −.04 | 1.0 (0.9-1.0) | <.001 |
| Very bad | −.22 | 0.8 (0.8-0.8) | <.001 |
| Chronic and long-term diseases lasting 6 months or longer |  |  |  |
| Yes | .57 | 1.8 (1.8-1.8) | <.001 |
| No | Reference |  |  |
| Hypertension |  |  |  |
| Yes | .18 | 1.2 (1.2-1.2) | <.001 |
| No | Reference |  |  |
| Diabetes |  |  |  |
| Yes | .19 | 1.2 (1.2-1.2) | <.001 |
| No | Reference |  |  |
| Heart diseases |  |  |  |
| Yes | .08 | 1.1 (1.1-1.1) | <.001 |
| No | Reference |  |  |
| COPDb |  |  |  |
| Yes | .11 | 1.1 (1.1-1.1) | <.001 |
| No | Reference |  |  |
| Allergy or asthma |  |  |  |
| Yes | .12 | 1.1 (1.1-1.1) | <.001 |
| No | Reference |  |  |
| Depression diagnosis |  |  |  |
| Yes | .15 | 1.2 (1.1-1.2) | <.001 |
| No | Reference |  |  |
| Cancer |  |  |  |
| Yes | .15 | 1.2 (1.1-1.2) | <.001 |
| No | Reference |  |  |
| Arthritis |  |  |  |
| Yes | .08 | 1.1 (1.1-1.1) | <.001 |
| No | Reference |  |  |
| Neurological disease |  |  |  |
| Yes | .19 | 1.2 (1.2-1.2) | <.001 |
| No | Reference |  |  |
| COVID-19 |  |  |  |
| Yes | .36 | 1.4 (1.4-1.4) | <.001 |
| No | Reference |  |  |
| Give up from financial reasons from a visit to the doctor |  |  |  |
| Yes | .09 | 1.1 (1.1-1.1) | <.001 |
| No | Reference |  |  |
| Have never been put in this situation | −.08 | 0.9 (0.9-0.9) | <.001 |
| Give up from financial reasons from a dental visit |  |  |  |
| Yes | .09 | 1.1 (1.1-1.1) | <.001 |
| No | Reference |  |  |
| Have never been put in this situation | .07 | 1.1 (1.1-1.1) | <.001 |
| Give up from financial reasons from purchasing a prescription drug |  |  |  |
| Yes | .15 | 1.2 (1.1-1.2) | <.001 |
| No | Reference |  |  |
| Have never been put in this situation | −.12 | 0.9 (0.9-0.9) | <.001 |
| Give up from financial reasons from purchasing needed medical equipment |  |  |  |
| Yes | .16 | 1.2 (1.2-1.2) | <.001 |
| No | Reference |  |  |
| Have never been put in this situation | .02 | 1.0 (1.0-1.0) | <.001 |
| Opportunity to provide transportation to the medical appointment |  |  |  |
| Yes, without any difficulty | .14 | 1.2 (1.1-1.2) | <.001 |
| Yes, but with some difficulties | .26 | 1.3 (1.3-1.3) | <.001 |
| Yes, but with great difficulties | .20 | 1.2 (1.2-1.3) | <.001 |
| Not able to provide transportation to the place of medical appointment | .06 | 1.1 (1.0-1.1) | .02 |
| Do not need transportation to the place of medical appointment—I walk there | Reference |  |  |
| Difficult to say | −.17 | 0.8 (0.8-0.9) | <.001 |
